# Perioperative statin therapy in cardiac and non-cardiac surgery: a systematic review and meta-analysis of randomized controlled trials

**DOI:** 10.1186/s13613-018-0441-3

**Published:** 2018-09-27

**Authors:** Alessandro Putzu, Carolina Maria Pinto Domingues de Carvalho e Silva, Juliano Pinheiro de Almeida, Alessandro Belletti, Tiziano Cassina, Giovanni Landoni, Ludhmila Abrahao Hajjar

**Affiliations:** 1 Division of Anesthesiology, Department of Anesthesiology, Pharmacology and Intensive Care Medicine, Geneva University Hospitals, Geneva, Switzerland; 2 Division of Anesthesia and Intensive Care, InCor, Instituto do Cancer, Universidade de Sao Paulo, São Paulo, Brazil; 3 Department of Anesthesia and Intensive Care, IRCCS San Raffaele Scientific Institute, Milan, Italy; 4 Department of Cardiovascular Anesthesia and Intensive Care, Cardiocentro Ticino, Lugano, Switzerland; 5 Vita-Salute San Raffaele University, Milan, Italy; 6 Department of Cardiopneumology, InCor, Universidade de Sao Paulo, São Paulo, Brazil

**Keywords:** Cardiac surgery, Intensive care medicine, Mortality, Non-cardiac surgery, Statins

## Abstract

**Background:**

The effects of perioperative statin therapy on clinical outcome after cardiac or non-cardiac surgery are controversial. We aimed to assess the association between perioperative statin therapy and postoperative outcome.

**Methods:**

Electronic databases were searched up to May 1, 2018, for randomized controlled trials of perioperative statin therapy versus placebo or no treatment in adult cardiac or non-cardiac surgery. Postoperative outcomes were: myocardial infarction, stroke, acute kidney injury (AKI), and mortality. We calculated risk ratio (RR) or odds ratio (OR) and 95% confidence interval (CI) using fixed-effects meta-analyses. We performed meta-regression and subgroup analyses to assess the possible influence of statin therapy regimen on clinical outcomes and trial sequential analysis to evaluate the risk of random errors and futility.

**Results:**

We included data from 35 RCTs involving 8200 patients. Perioperative statin therapy was associated with lower incidence of postoperative myocardial infarction in non-cardiac surgery (OR = 0.44 [95% CI 0.30–0.64], *p *< 0.0001), but not in cardiac surgery (OR = 0.93 [95% CI 0.70–1.24], *p *= 0.61) (*p*_*subgroup*_ = 0.002). Higher incidence of AKI was present in cardiac surgery patients receiving perioperative statins (RR = 1.15 [95% CI 1.00–1.31], *p *= 0.05), nonetheless not in non-cardiac surgery (RR = 1.52 [95% CI 0.71–3.26], *p *= 0.28) (*p*_*subgroup*_ = 0.47). No difference in postoperative stroke and mortality was present in either cardiac or non-cardiac surgery. However, low risk of bias trials performed in cardiac surgery showed a higher mortality with statins versus placebo (OR = 3.71 [95% CI 1.03–13.34], *p *= 0.04). Subgroup and meta-regression analyses failed to find possible relationships between length of statin regimens and clinical outcomes. Trial sequential analysis suggested no firm conclusions on the topic.

**Conclusions:**

Perioperative statins appear to be protective against postoperative myocardial infarction in non-cardiac surgery and associated with higher AKI in cardiac surgery. Possible positive or even negative effects on mortality could not be excluded and merits further investigations. Currently, no randomized evidence supports the systematic administration of statins in surgical patients.

**Electronic supplementary material:**

The online version of this article (10.1186/s13613-018-0441-3) contains supplementary material, which is available to authorized users.

## Background

Perioperative complications are still relatively frequent in patients undergoing cardiac or non-cardiac surgery, and attempts to minimize complications and deaths are crucial. Pathophysiological mechanisms behind clinical complications may include inflammatory processes after surgery.

The knowledge of statin’s pleiotropic and anti-inflammatory effects has led to consider perioperative statin a potential treatment able to modulate the clinical outcome after surgery [1]. Previous studies suggested beneficial effects of perioperative statin therapy on postoperative outcome after high-risk non-cardiac surgery [2–4], with a previous meta-analysis advising that perioperative statin therapy decreases the perioperative incidence of mortality and myocardial infarction in this high-risk population [5].

In contrast, growing evidences on perioperative statins administration in cardiac surgery suggested neutral or even detrimental effects. In facts, two large, high-quality, trials randomizing cardiac surgery patients to receive perioperative statin or placebo were recently published [6, 7]. Perioperative statins did not prevent postoperative atrial fibrillation or perioperative myocardial damage, but acute kidney injury (AKI) was more common in patients receiving statin. All this evidence supported a potential neutral or even negative effect of perioperative statin in cardiac surgery.

We conducted a systematic review and meta-analysis of randomized controlled trials (RCTs) to provide clinical guidance of administration of perioperative statin therapy in the perioperative period. We aimed to assess the effects on postoperative myocardial infarction, stroke, AKI, and mortality in adult cardiac and non-cardiac surgery in patients treated with perioperative statins.

## Methods

We performed a systematic review and meta-analysis of RCTs, in compliance with Preferred Reporting Items for Systematic Reviews and Meta-Analyses (PRISMA) guidelines [8] and the Cochrane methodology [9], and according to a pre-published protocol (PROSPERO database, CRD42018093997) [10]. A PRISMA checklist is available in the supplement (eTable 1, Additional file 1). The authors had no conflicts of interests.

### Search strategy

Two trained investigators (AP and CS) independently searched PubMed, the Cochrane Central Register of clinical trials, and EMBASE (last updated on May 1, 2018) for appropriate articles. The search strategy for PubMed is reported in the supplement (eMethods 1, Additional file 1) and was designed to include any RCT ever published with perioperative statin therapy compared to control in adult humans undergoing surgery. In addition, we employed backward snowballing (i.e., scanning of references of pertinent articles). No language restriction was enforced.

### Study selection

References, obtained from database and literature searches, were examined first at an abstract level independently by 2 investigators (AP and CS), with eventual divergences resolved by consensus, and then, if potentially pertinent, were retrieved as complete articles. Eligible studies met the following PICOS criteria: (1) Population: adult cardiac and non-cardiac surgery patients; (2) Intervention: perioperative administration of statin therapy; (3) Comparison intervention: placebo or no active intervention as control; (4) Outcome: any outcome of the present systematic review (see below); (5) Study design: RCT. The exclusion criteria were: overlapping populations and pediatric studies. Two authors (AP and JP or AB) independently assessed selected studies for the analysis, with divergences resolved by consensus with a third author (GL).

### Data abstraction and study endpoint

Baseline characteristics, procedural, and outcome data were abstracted by one author (AP) extracted relevant information from each selected study, and these data were checked by a second author (JP or AB). Specifically, we extracted potential sources of significant clinical heterogeneity, such as study design, clinical setting/indication, statin dose, and control treatment, as well as primary study outcomes.

The per-protocol primary outcomes were: postoperative MI, postoperative stroke, postoperative AKI, and mortality at the longest follow-up available. Post hoc secondary outcomes were AKI requiring postoperative renal replacement therapy (RRT) and AKI not requiring RRT. The outcomes were reported as per-author definition. We extracted data following the intention-to-treat basis whenever possible. Corresponding authors of all eligible articles were contacted in case of missing data on outcomes of interest.

### Risk of bias assessment

We used the Cochrane methodology to evaluate the methodological quality of each included trial [9]. Each trial was finally judged to be of low, unclear, or high risk of bias (eMethods 2, Additional file 1). Publication bias was assessed by visually inspecting funnel plots for pooled analyses containing > 10 studies [9].

### Statistical analysis

The analysis was stratified according to cardiac or non-cardiac surgery setting. For each outcome, we calculated the odds ratio (OR) with 95% confidence intervals (CI). For common events, pre-defined as frequency of the event in the control group > 10%, we calculated risk ratio (RR) with 95% CI [9]. A *p* value equal or less than 0.05 was considered significant. In case of statistical significant results, we calculated the number needed to treat (NNT) or number needed to harm (NNH) and 95% CI. Heterogeneity was explored by the Cochran *Q* statistic and characterized with *I*^2^. We used a fixed-effects model in the absence of significant heterogeneity, defined as *p* value > 0.10 and *I*^2^ < 50%. In case of significant heterogeneity, we employed the random-effects model except if few trials dominate the available evidence or if significant publication bias was present, since random-effects meta-analysis, in these contexts, can give inappropriate high weight to smaller studies [9].

According to Cochrane methodology [9], we performed subgroup analyses for each outcome in order to assess the influence of trials’ risk of bias, including only low risk of bias trials or only trials with unclear or high risk of bias. Sensitivity analyses were performed changing summary statistic (OR, RR, risk difference) and according to possible conflicts of interests/funding. Meta-regression was employed to examine the possible influence of statin therapy regimen on clinical outcomes. Subgroup analyses were performed on trials that included only statin-naïve patients or trials not employing only statin-naïve patients (trials enrolling a population of chronic statin therapy or a mixed population). Subgroup differences were tested using Chi-square statistics [9]. The meta-analysis was performed using Review Manager (RevMan [Computer program], version 5.3. Copenhagen: The Nordic Cochrane Centre, The Cochrane Collaboration, 2014). All the analyses were pre-defined [10].

### Trial sequential analysis

To control risks of random errors due to sparse data and repetitive testing of cumulative data, we performed a per-protocol fixed-effects trial sequential analysis (TSA). TSA is a methodology that combines an information size calculation, representative of the cumulated sample sizes of all included trials, with a threshold for a statistically significant treatment effect and a threshold for futility of the intervention. In particular, TSA pools the required information size with trial sequential monitoring boundaries which adjust the confidence intervals and decrease type I errors [11–13]. In TSA, the inclusion of each trial in the meta-analysis is regarded as an interim meta-analysis and TSA permits to control the risk for type I and type II errors and helps to clarify whether additional trials are needed. Conclusions made using TSA show the potential to be more consistent than those using traditional meta-analysis techniques [11–13]. We conducted TSA with the purpose to maintain an overall 5% risk of type I error and a 10% risk of type II error, at a power of 90%. We assumed a relative risk reduction (RRR) or relative risk increase (RRI) of 15%, and we derived the control event proportion from low risk of bias trial. The resulting required information size was further diversity (*D*^2^)-adjusted; in case of *D*^2^ = 0, we performed a sensitivity analysis assuming a *D*^2^ = 25%. We used the TSA software (TSA Viewer [Computer program], version 0.9.5.5 Beta, Copenhagen Trial Unit, Centre for Clinical Intervention Research, Rigshospitalet, 2016).

## Results

### Study characteristics

**Fig. 1:**
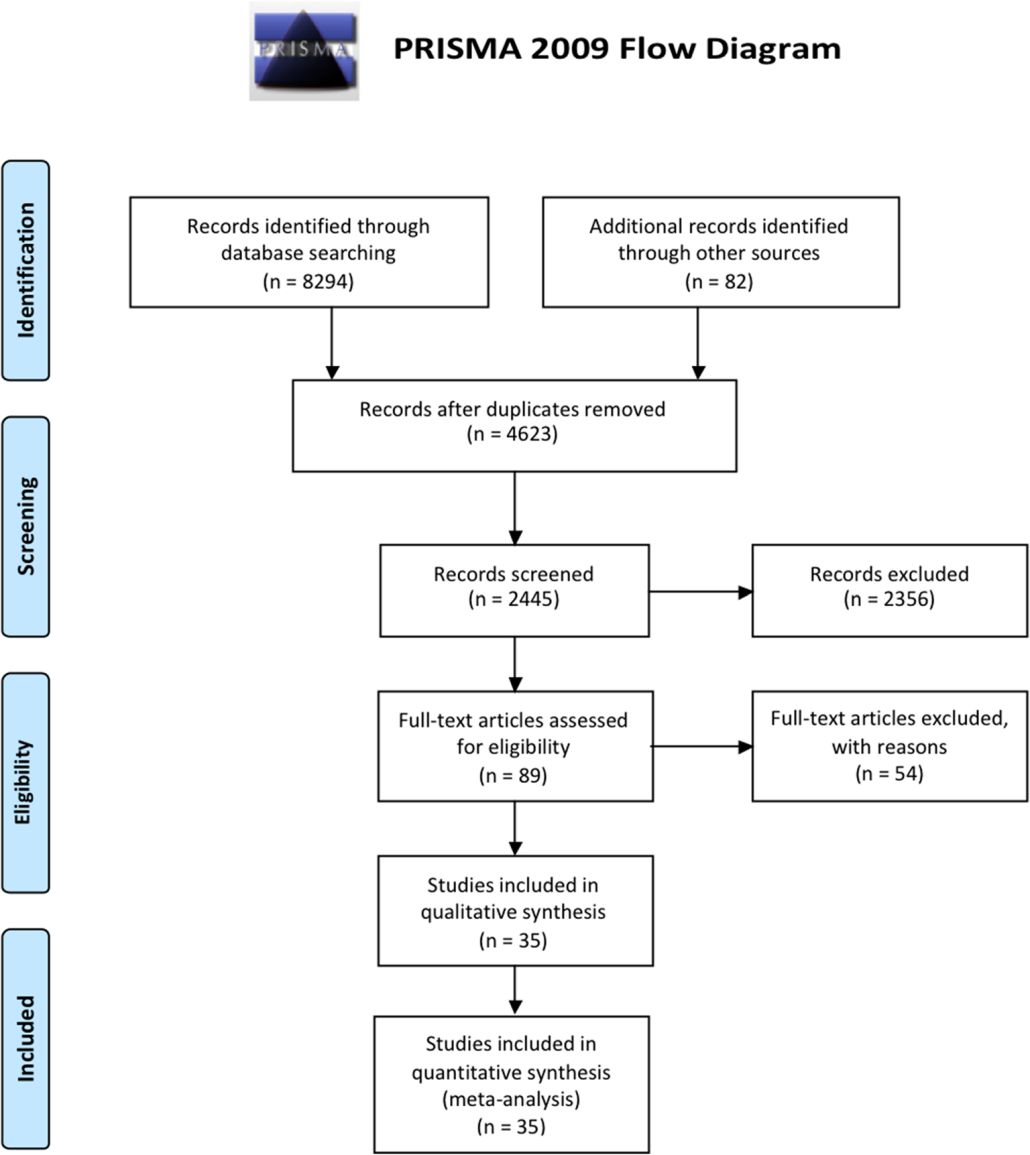
Study flow diagram

The literature search yielded 8376 references of which 35 RCTs (8200 randomized patients) [2, 3, 6, 7, 14–44] met the eligibility criteria and were included in the analysis (Fig. 1). Major exclusions are presented in the supplement (eTable 2, Additional file 1). Characteristics of the included trials are reported in Table 1 and in eTable 3 in the Additional file 1.

**Table 1:** Characteristics of the trials included in the analysis

Trial	Journal	Surgical procedure	Number of patients	Statin	Statin regimen	Control	Patients naïve to statin therapy (%)	Outcomes for meta-analysis	Risk of bias
*Cardiac surgery*									
Almansob 2012	Arterioscler Thromb Vasc Biol	Non-coronary cardiac surgery	132	Simvastatin 20 mg	5–7 days pre-op and post-op day 2	No treatment	nr	MI, S, M	High
Aydin 2015	Anatol J Cardiol	On-pump CABG	60	Atorvastatin	7 h post-op and 30 days post-op	No treatment	100	MI, M	High
Baran 2011	Stem Cell Rev and Rep	On-pump CABG	60	Atorvastatin 40 mg	14 days pre-op and post-op (no day 0)	Placebo	100	AKI, MI, S, M, RRT	High
Berkan 2008	Thorac Cardiov Surg	On-pump CABG	46	Fluvastatin 80 mg	3 weeks pre-op	Placebo	100	MI	High
Billings 2016	JAMA	On-/Off-pump cardiac surgery	615	Atorvastatin 40 mg	Bid day 1, qd day 0, and qd post-op^°^	Placebo	32	AKI, MI, S, M, RRT	Low
Carrascal 2016	Journal of Arrhythmia	Valvular surgery	90	Atorvastatin 40 mg	7 days pre-op and 7 days post-op	No treatment	100	AKI, MI, S, M, RRT	High
Castaño 2015	J Cardiovasc Surg	On-pump CABG	30	Pravastatin 40 or 80 mg	2 h pre-op	Placebo	0	AKI, MI, S, M, RRT	Low
Chello 2006	Crit Care Med	On-pump CABG	40	Atorvastatin 20 mg	3 weeks pre-op	Placebo	100	AKI, MI, S, M	Unclear
Christenson 1998	Eur J Cardiothorac Surg	On-pump CABG	77	Simvastatin 20 mg	4 weeks pre-op	No treatment	nr	AKI, MI, M	High
Dehghani 2014	J Cardiovasc Pharmacol Ther	Valvular surgery	58	Atorvastatin 40 mg	3 days pre-op and 5 days post-op	Placebo	100	MI, S, M	High
Hua 2017	Biomed Res Int	Valvular surgery	130	Simvastatin 20 mg	5–7 days pre-op and 7 days post-op (no days 0–1).	Placebo	100	M	High
Ji 2009	Circ J	Off-pump CABG	140	Atorvastatin 20 mg	7 days pre-op	Placebo	100	MI, S, M	High
Mannacio 2008	J Thorac Cardiovasc Surg	On-pump CABG	200	Rosuvastatin 20 mg	7 days pre-op	Placebo	100	AKI, MI, S, M	High
Mansour 2016	Int J Adv Biomed	Cardiac surgery	50	Atorvastatin 40 mg	7 days pre-op and post-op	No treatment	100	MI, S, M	High
Nakamura 2006	Cytokine	On-/Off-pump CABG	31	Atorvastatin 10 mg	Unclear	No treatment	32	M	Unclear
Park 2016	Intensive Care Med	Valvular surgery	200	Atorvastatin 40 mg	Bid day − 1 and qd days 0–1–2	Placebo	100	AKI, MI, S, M, RRT	Low
Patti 2006	Circulation	On-pump cardiac surgery	200	Atorvastatin 40 mg	7 days pre-op and post-op until discharge (no day 0)	Placebo	100	MI, M	High
Prowle 2012	Nephrology	On-pump cardiac surgery	100	Atorvastatin 40 mg	Days 0–1–2–3	Placebo	30	AKI, M, RRT	Low
Song 2008	Am Heart J	Off-pump CABG	124	Atorvastatin 20 mg	3 days pre-op and continued for 30 days post-op	No treatment	100	MI, S	High
Spadaccio 2010	J Cardiovasc Pharmacol	On-pump CABG	50	Atorvastatin 20 mg	3 weeks pre-op	Placebo	100	AKI, MI, S, M	Unclear
Sun 2011	Int Heart J	On-pump CABG	100	Atorvastatin 20 mg	7 days pre-op	Placebo	100	MI	High
Tamayo 2009	J Thorac Cardiovasc Surg	On-pump CABG	44	Simvastatin 20 mg	3 weeks pre-op	No treatment	100	M	High
Vukovic 2010	Perfusion	On-pump CABG	57	Atorvastatin 20 mg	3 weeks pre-op	Placebo	100	MI, M	High
Youn 2011	Korean J Thorac Cardiovasc Surg	Off-pump CABG	142	Rosuvastatin 40 mg	Bid day 1, qd day 0	No treatment	45	MI, M	High
Zheng 2016	N Engl J Med	On–/Off-pump CABG/AVR/CABG + AVR	1922	Rosuvastatin 20 mg	1–8 days pre-op and 5 days post-op	Placebo	66	AKI, MI, S, M, RRT	Low
*Non*-*cardiac surgery*									
Amar 2015	J Thorac Cardiovasc Surg	Pulmonary resection	88	Atorvastatin 40 mg	7 days pre-op and 7 days post-op	Placebo	100	MI, M	High
Bass 2018	HSSJ	Hip fracture and total hip/knee replacement surgery	20	Atorvastatin 40 mg	4 days pre-op and 45 days post-op	Placebo	100	AKI, MI, S, M	High
Berwanger 2017	Am Heart J	Non-cardiac surgery in high-risk patients^a^	642	Atorvastatin 80 mg	1 day pre-op and seven days post-op	Placebo	100	AKI, MI, S, M	High
Durazzo 2004	J Vasc Surg	Aortic, femoropopliteal, and carotid vascular surgery	100	Atorvastatin 20 mg	At least 14 days pre-op and up to 4 weeks post-op	Placebo	100	MI, S, M	Low
Neilipovitz 2012	Can J Anaesth	Non-cardiac surgery in high-risk patients^b^	76	Atorvastatin 80 mg	7 days pre-op and 7 days post-op or day 0 and 7 days post-op	Placebo	100	MI, S, M	High
Parepa 2017	Farmacia	Elective non-cardiac, non- vascular surgery without known cardiac disease	1380	Rosuvastatin 10 mg	10 days pre-op and 20 days post-op	Placebo	100	MI, M	High
Shyamsundar 2014	Annals of Surgery	Esophagectomy	31	Simvastatin 80 mg	4 days pre-op and 7 days post-op	Placebo	100	AKI, MI, S, M	Unclear
Singh 2016	J Am Coll Surg	Colorectal resection or reversal of Hartmann’s procedure surgery	132	Simvastatin 40 mg	3–7 days pre-op and 14 days post-op	Placebo	100	M	Low
Xia 2014	Cardiology	Urgent abdominal surgery^c^ in patients with stable CAD	500	Atorvastatin 80 mg	2 h before surgery	Placebo	0	MI, M	Low
Xia 2015	Cardiology	Urgent abdominal surgery^c^ in patients with stable CAD	550	Rosuvastatin 20 mg	2 h before surgery	Placebo	0	MI, S, M	Unclear

AKI, acute kidney injury; AVR, aortic valve replacement; CABG, coronary artery bypass grafting; CAD, coronary artery disease; M, mortality; MI, myocardial infarction; RRT, renal replacement therapy; nr, not reported; pre-op, pre-operatively; post-op, post-operatively; day 0, the morning of the day of surgery; qd, once a day; bid, twice a day; °, patients on chronic statin therapy received study drug only on day 0 and day 1, resuming chronic statin therapy on postoperative day 2

^a^Defined as: history of CAD, peripheral vascular disease, stroke, major vascular surgery, or any 3 of 7 risk factors criteria (intrathoracic or intraperitoneal surgery, congestive heart failure, transient ischemic attack, diabetes, renal failure, age > 70 years, or emergent/urgent surgery)

^b^Defined as: history of CAD, peripheral vascular disease, stroke, or congestive heart failure, or three of six risk factor (high-risk surgery, previous congestive heart failure, diabetes, renal failure, age > 70 years, previous transient ischemic attack)

^c^Acute suppurative appendicitis, acute cholecystitis, acute cholangitis, acute pancreatitis, peptic ulcer perforation or urinary calculi

Twenty-five trials (4698 patients) included elective cardiac surgery patients, with coronary artery bypass grafting surgery as the most represented procedure, performed in 72.29% of the patients. Ten trials (3502 patients) were performed in non-cardiac surgery setting [2, 3, 17, 19, 29, 33, 34, 39, 41, 43], in particular: 2 in elective non-cardiac surgery, 2 trials in elective vascular surgery, 1 in elective lung resection surgery, 1 in elective orthopedic surgery, 1 in elective esophagectomy, 1 in elective colorectal surgery, and 2 in urgent abdominal surgery.

Twenty-four trails included exclusively statin-naïve patients (4101 patients), 3 trials included patients on chronic statin therapy (1080 patients), and 5 trials had a mixed population (2810 patients). All trials administered statins preoperatively and 19 trials postoperatively. Length of preoperative statin treatment ranged from 1 to 28 days (median 7 days), and the total duration of therapy varied from 2 to 45 days (median 7 days). Atorvastatin (10–80 mg) was administered in 22 trials, simvastatin (20–80 mg) in 6 trials, rosuvastatin (10 and 20 mg) in 5 trials, pravastatin (40 or 80 mg) in 1 trial, and fluvastatin 80 mg in 1 trial. Twenty-six trials administered placebo as control, and 9 trials administered no intervention as control.

**Fig. 2:**
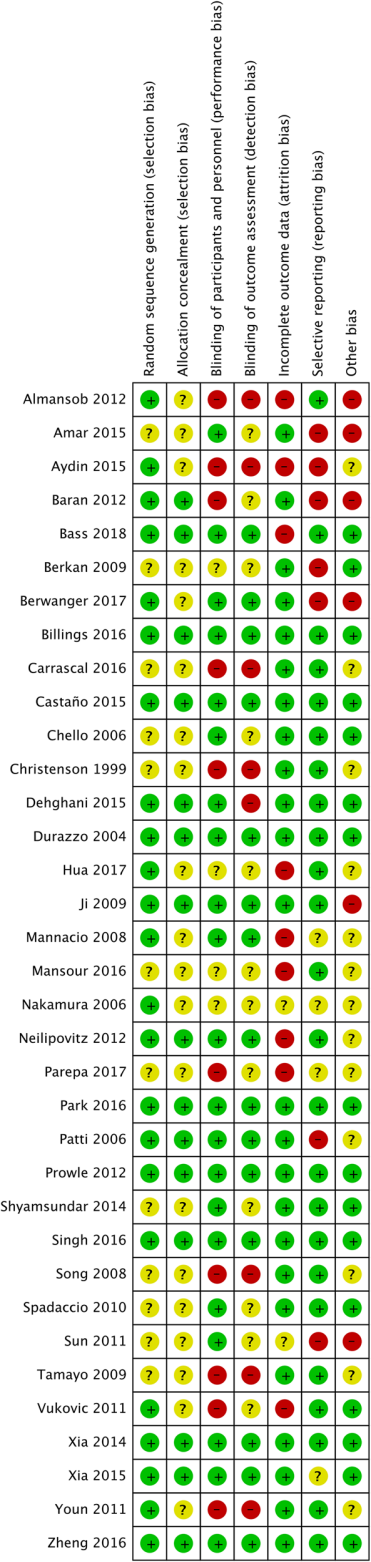
Risk of bias summary: review authors’ judgments about each risk of bias item for each included study

Eight trials were judged to be at low risk of bias in all bias domains [3, 6, 7, 21, 30, 32, 34, 39]. Five trials scored unclear-risk of bias [22, 33, 35, 43, 44], and 22 trials were at high risk of bias (Fig. 2 and eFigure 1, Additional file 1). No publication bias was found (eFigure 2–5, Additional file 1). Sensitivity analysis according to conflicts of interest or funding was consistent with primary analysis (eResults 1, Additional file 1).

In 13 cases, we received further outcomes’ data from the authors [6, 14, 17, 19–22, 24, 27, 30, 32–34].

### Myocardial infarction

The rate of postoperative MI was lower in non-cardiac surgery patients randomized to statins (OR = 0.44 [95% CI 0.30–0.64], NNT = 36 [95% CI 25–66]). However, TSA was not conclusive for a RRR = 15% (OR = 0.44 [TSA-adjusted 95% CI 0.16–1.19], 18.27% of the information size accrued) suggesting the need of further trials for a firm conclusion (eFigure 6, Additional file 1). The results were similar when limiting the analysis to low risk of bias trials (OR = 0.28 [95% CI 0.13–0.64], 2 trials and 300 patients) and at sensitivity analyses (eTables 4 and 5, Additional file 1).

**Fig. 3:**
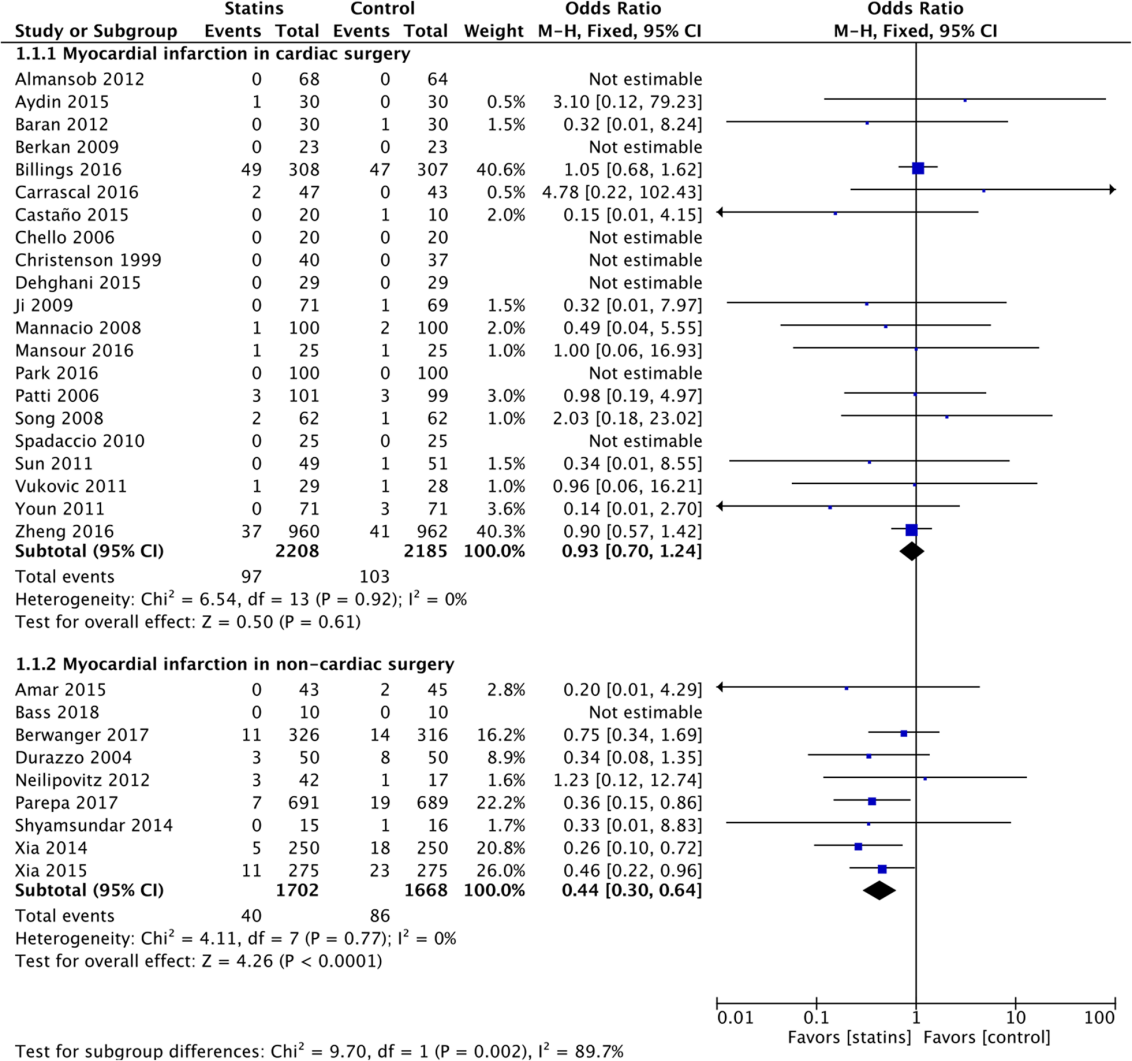
Postoperative myocardial infarction (MI). Forest plot for postoperative MI in patients with perioperative statin therapy versus control

In contrast, no beneficial effects related to statin administration were found in cardiac surgery (OR = 0.93 [95% CI 0.70–1.24], TSA inconclusive) (*p*_subgroup_ = 0.002) (Fig. 3), with results consistent at secondary analyses (eTables 4 and 5, Additional file 1).

### Stroke

**Fig. 4:**
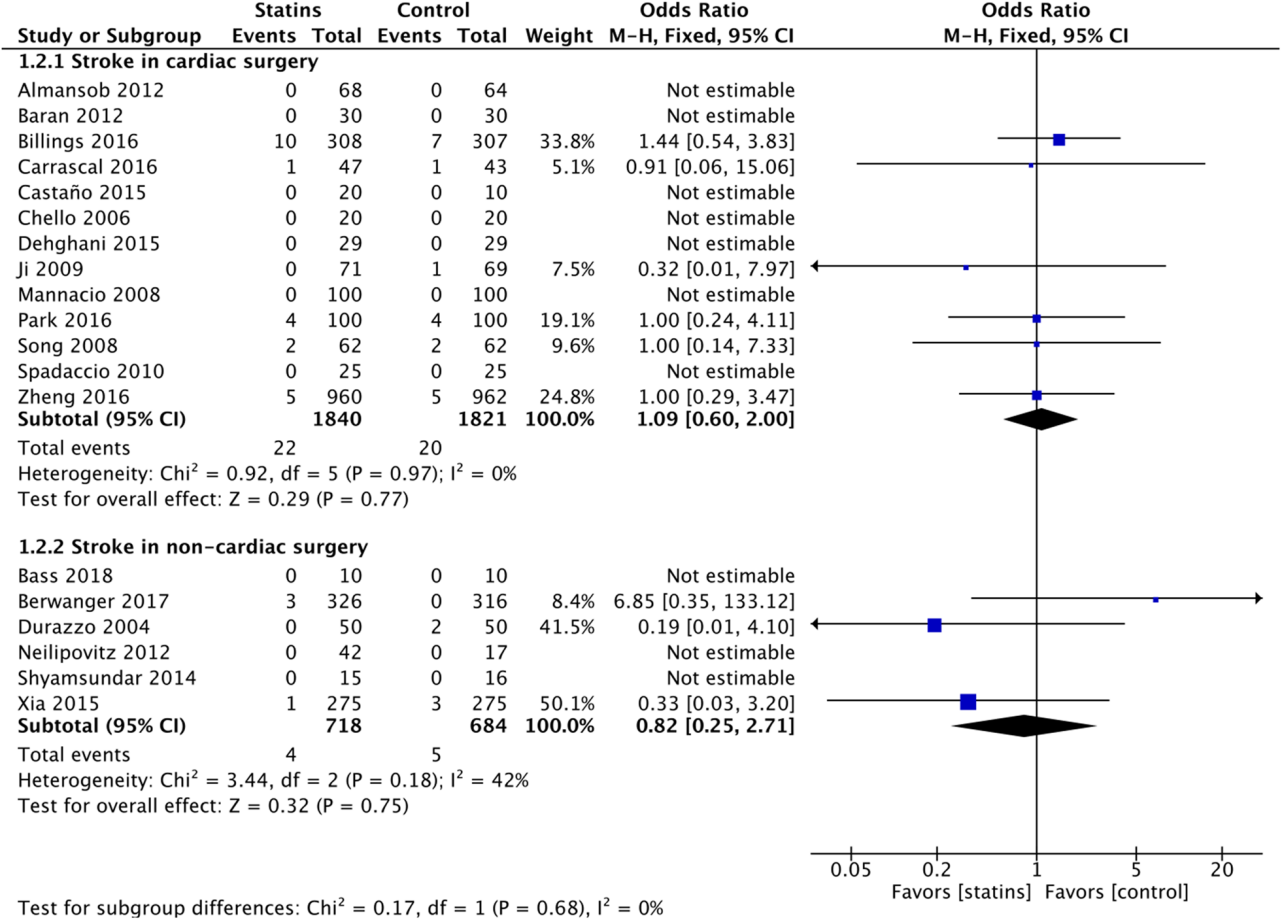
Postoperative stroke. Forest plot for postoperative stroke in patients with perioperative statin therapy versus control

No significant difference in postoperative stroke rate was found neither in cardiac surgery (OR = 1.10 [95% CI 0.60–2.03]) nor in non-cardiac surgery (OR = 0.71 [95% CI 0.09–5.64]) (Fig. 4), with TSA inconclusive and similar results at secondary analyses (eTable 4, Additional file 1).

### Acute kidney injury and renal replacement therapy

**Fig. 5:**
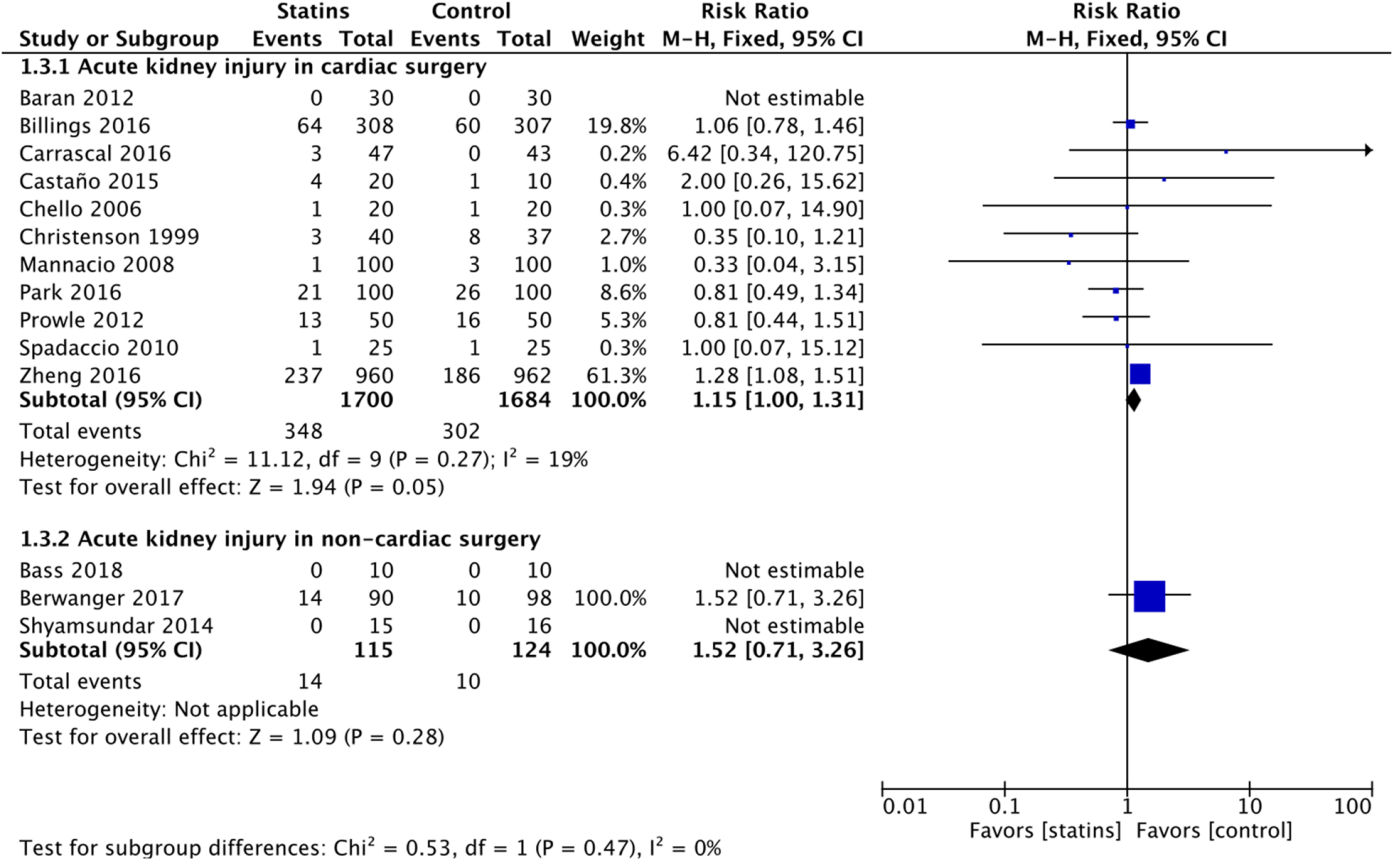
Postoperative acute kidney injury (AKI). Forest plot for postoperative AKI in patients with perioperative statin therapy versus control

Perioperative statins were associated with higher incidence of AKI when compared to control in cardiac surgery patients (11 trials and 3384 patients, RR = 1.15 [95% CI 1.00–1.31], NNH = 40 [95% CI NNH = 19 to NNT = 866]) (Fig. 5), also when limiting the analysis to low risk of bias trials (1.17 [95% CI 1.02–1.34], NNH = 30 [95% CI 16–307]) (eTable 4, Additional file 1). At sensitivity analysis, the effect was not evident when employing a random-effects model (all trials: RR = 1.05 [95% CI 0.85–1.30]) or when limiting the analysis to statin-naïve patients (RR = 1.09 [95% CI 0.75–1.57]). Trial sequential analysis was inconclusive for a RRI = 15% due to the too small cumulative information size (eTable 4, Additional file 1).

No significant effect was found in non-cardiac surgery population (RR = 1.52 [95% CI 0.71–3.26], TSA inconclusive) (Fig. 4), but only 3 trials (239 patients) reported the outcome, with all the events coming from 1 trial [19].

In cardiac surgery, no significant difference in postoperative RRT was found (OR = 1.46 [95% CI 0.75–2.81]), while AKI not requiring RRT was higher in the statin group (OR = 1.22 [95% CI 1.02–1.46]) (*p*_groups_ = 0.61) (eTable 4, Additional file 1). No trials performed in non-cardiac surgery reported these outcomes.

### Mortality

**Fig. 6:**
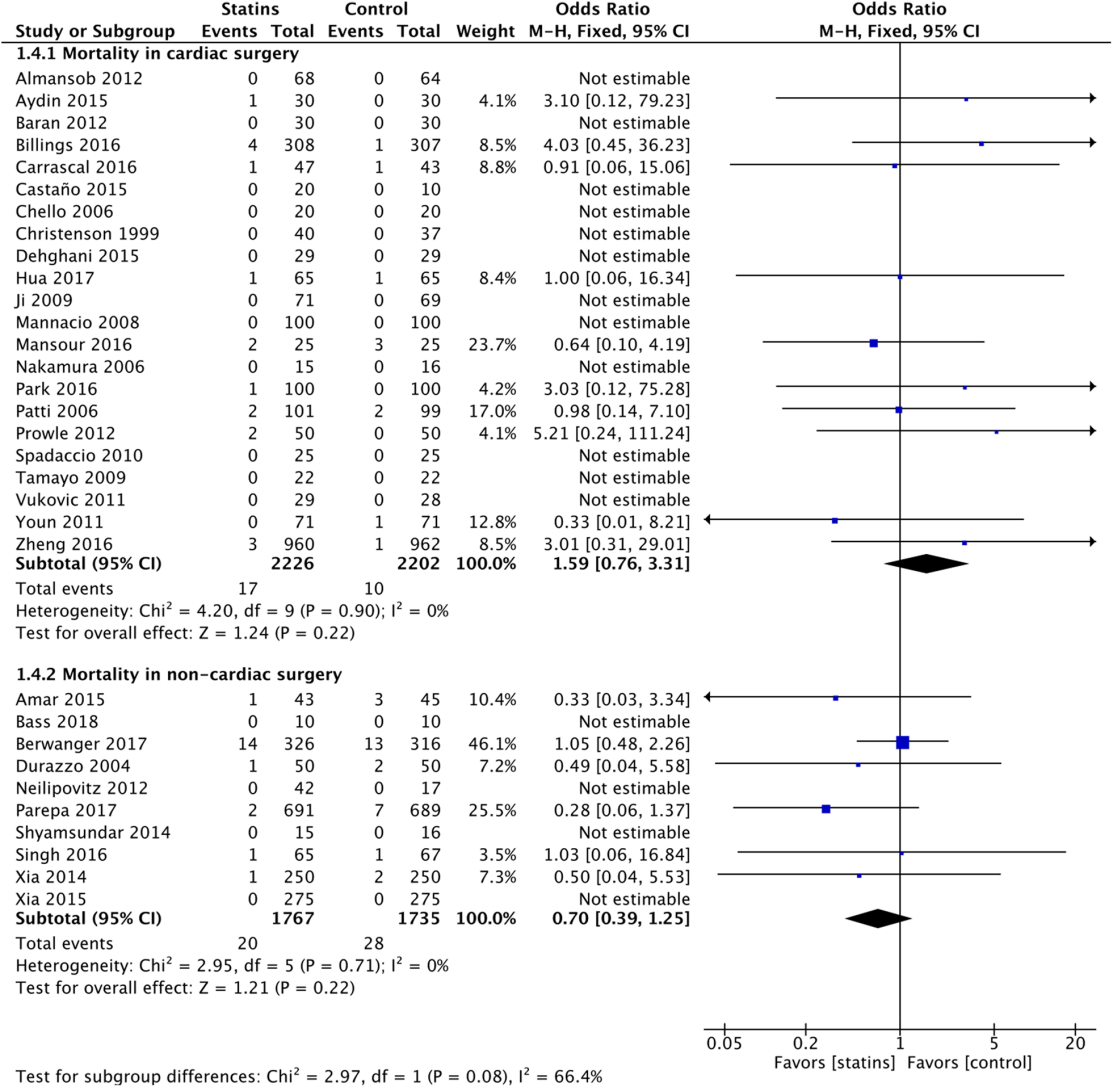
Postoperative mortality. Forest plot for postoperative short-term mortality in patients with perioperative statin therapy versus control (the longest follow-up available, median 30 days)

The administration of perioperative statin therapy was associated with no significant difference in mortality in both study population, at a median follow-up of 30-days (Fig. 6). However, when assessing only lower risk of bias trials, perioperative statins were associated with increased mortality in the cardiac surgery population (OR = 3.71 [95% CI 1.03–13.34], NNH = 181 [95% CI 97–1187]) (eTable 4, Additional file 1). Trial sequential analysis suggested no firm evidence for a RRI = 15% and the need of further randomized trials.

### Clinical outcomes and statin regimen

In statin-naïve patients undergoing cardiac surgery, perioperative statins are associated with no significant differences in all the assessed outcomes (eTable 6, Additional file 1). Trials enrolling chronic statin users or a mixed population showed that perioperative statins are associated with a higher risk of AKI in cardiac surgery (RR = 1.16 [95% CI 1.00–1.34]) and no differences in other outcomes (eTable 6, Additional file 1).

In non-cardiac surgery, lower myocardial infarction is evident in both statin-naïve (OR = 0.49 [95% CI 0.30–0.81]) and chronic statin patients (OR = 0.37 [95% CI 0.21–0.67]), and no differences in other outcomes (eTable 6, Additional file 1). No significant between-groups differences were found between statin-naïve trials and other trials enrolling patients on chronic statin therapy or a mixed population (*p*_groups_ > 0.05) (eTable 6, Additional file 1).

Meta-regression analysis failed to find possible relationships between length of pre- or postoperative statin regimen and clinical outcomes (eResults 2, Additional file 1).

## Discussion

Our systematic review and meta-analysis suggests that perioperative statin therapy could be protective against postoperative myocardial infarction in non-cardiac surgery but associated with an increased risk of acute kidney injury in cardiac surgery. Statins were associated with an increase in hospital mortality in cardiac surgery in low risk of bias trials. However, the quality and quantity of randomized evidence are still insufficient to allow a firm conclusion on the topic.

This is the largest and most comprehensive meta-analysis of RCTs of statins in surgery performed so far, including 35 randomized trials and 8200 patients, evaluating the effect of statin therapy on outcomes in both non-cardiac and cardiac surgery using separate analysis. In non-cardiac surgery, a previous meta-analysis that evaluated 5 RCTs in vascular surgery did not show any detrimental or favorable effect of statins on postoperative outcomes [45]. De Waal et al., in a meta-analysis of 16 trials, only included statin-naïve patients undergoing surgery and showed that starting statin therapy, in this cohort of patients who were not already on long-term statin treatment, reduces perioperative mortality, myocardial infarction, atrial fibrillation, and decreases length of hospital stay [5]. Our study is consistent with previous findings regarding prevention in postoperative myocardial infarction, but we failed to find any other possible beneficial effects, even in the statin-naïve subgroup. On the other hand, a recent meta-analysis evaluated 23 trials in cardiac surgery and showed no effect of statin on postoperative incidence of myocardial infarction, infection, stroke, and atrial fibrillation, and a higher occurrence of AKI [46].

A potential beneficial effect of perioperative statins on postoperative MI in non-cardiac surgery was found, in contraposition to the lack of effects in cardiac surgery patients. Regarding non-cardiac surgery, a randomized study suggested that a short-term treatment with atorvastatin significantly reduces the incidence of major adverse cardiovascular events after vascular surgery [2]. In contrast, a more recent and larger trial found neutral results and did not demonstrate a reduction in major cardiovascular complications after a short-term perioperative course of statin in statin-naïve non-cardiac surgery patients, even in the vascular surgery subgroup [19]. Chopra and colleagues, in a meta-analysis published in 2012 including 15 RCTs with 2292 patients undergoing cardiac and non-cardiac surgeries, found that perioperative statin treatment reduced the risk of myocardial infarction [4]. Nowadays, the growing quality and quantity of data can allow a more thoughtful analysis, confirming the potential benefits of statin in non-cardiac surgery in terms of MI prevention, but not in cardiac surgery. In fact, in the largest RCTs performed so far in cardiac surgery patients, no difference was found in postoperative MI and myocardial injury [6, 7]. Perioperative myocardial ischemia in non-cardiac surgery occurs mostly as consequence of endogenous catecholamine release and acute inflammatory response, resulting in an increased oxygen demand, heart rate, and contractility. Inflammation also leads to changes in atheromatous plaque features, culminating in the rupture of lipid-laden vulnerable lesions, thrombocytes activation with development of platelet-rich thrombi, acute vessel occlusion, and ischemia [47, 48]. Perioperative statins might reduce the risk of perioperative infarction due to its properties of endothelial modulation resulting in vasodilation in addition to its anti-inflammatory effects, reducing plaque instability [49]. In cardiac surgery, myocardial infarction occurs mainly due to an ischemic event arising from either a failure in graft function, an acute coronary event involving the native coronary arteries, or inadequate cardioprotection during cardiopulmonary bypass. We might postulate that the different pathophysiology mechanisms could explain our findings of no protection of statins against MI after cardiac surgery. In addition, our findings showed no effect of perioperative statin in postoperative stroke, another crucial cardiovascular complication. However, further investigations are warranted in patients at high risk of cerebrovascular events.

Our systematic review, including 25 studies in cardiac surgery, of whom 5 with low risk of bias, confirms the previous findings of possible negative effects of statins on renal function of cardiac surgery patients. For many years, perioperative statins were considered an attractive therapy for preventing AKI following cardiac surgery, hypothesis based mainly on the positive results from retrospective series or high risk of bias RCTs [50]. Nowadays, high-quality RCTs support the lack of a kidney-protective effect [6, 7, 30]. The largest RCT showed that rosuvastatin therapy resulted in a significantly higher rate of AKI and plasma creatinine compared to placebo at 48 h after cardiac surgery [7]. Similarly, the second largest RCT published so far showed a non-significant trend in favor of placebo and a possible harmful effect of perioperative statins in the small subgroup of statin-naïve patients with chronic kidney disease [6]. The exact mechanisms of increased occurrence of AKI related to statin use need to be elucidated. There are possible effects of statin in kidney function, including mitochondrial dysfunction leading to overall cellular energy imbalance [50–52]. Further research is needed, especially in the non-cardiac surgery setting, where evidence is still limited, not allowing any conclusion on possible renal effects of statin in this population.

### Strengths and limitations

Our study has some limitations, which most of them are characteristics of all aggregate data meta-analyses [53]. Different statin dosages and formulations were used as statin therapy in the included studies. We did not perform subgroup analysis of different types of perioperative statin regimens, since most of the trials administered different statin dose and formulation for different length of time, and the analysis would be underpowered and mainly driven by results of small and higher risk of bias trials. Another limitation is that only few high-quality randomized trials have been published so far in non-cardiac surgery setting. To assess the effects of methodological biases on results [54], we assessed subgroup analyses according to trial risk of bias. A major strength of this meta-analysis is that we assessed the most important clinical outcome, mortality. We decided not to assess surrogate outcomes commonly related to statin administration, such as myalgia or creatinine kinase elevation, due to the heterogeneity in definitions and spurious reporting. Finally, most of the included trials were focused on perioperative period and did not report clinical outcomes at longer follow-up.

Further high-quality trials should systematically evaluate the relationship between postoperative outcomes and patients’ variables (e.g., statin-naïve, chronic kidney disease), to the surgery, and to the statin regimen (e.g., therapy duration).

## Conclusions

Our results suggest that perioperative statins appear to be protective against postoperative myocardial infarction in non-cardiac surgery and associated with an increased risk of acute kidney injury in cardiac surgery. There are still insufficient randomized data for firm conclusions on perioperative statin therapy, and possible positive or even negative effects on mortality could not be excluded. No randomized evidence supports the systematic administration of statins in surgical patients, especially in statin-naïve patients. Further RCTs should evaluate the safety profile, possible beneficial effects on patients’ outcome, particularly in non-cardiac surgery, and assess the more appropriate time-point for eventual statin discontinuation before surgery in patients under chronic statin therapy.

## Additional file

**Additional file 1: Table 1.** PRISMA 2009 checklist. **Table 2**. Major exclusions. **Table 3**. Further characteristics of the included trial. **Figure 1**. Risk of bias graph. **Figure 2**. Funnel plot for myocardial infarction. **Figure 3**. Funnel plot for stroke. **Figure 4**. Funnel plot for acute kidney injury. **Figure 5**. Funnel plot for mortality. **Figure 6**. Trial sequential analysis for postoperative myocardial infarction. **Table 4**. Primary and secondary analyses. **Table 5**. Sensitivity analyses. **Table 6**. Postoperative outcomes in statin-naïve trials.

## Abbreviations

AKI: acute kidney injury
CI: confidence interval
NNH: number needed to harm
NNT: number needed to treat
OR: odds ratio
RR: risk ratio
RRT: renal replacement therapy

## References

[1] Le Manach Y, Coriat P, Collard CD, Riedel B (2008). Statin therapy within the perioperative period. Anesthesiology.

[2] Amar D, Park B, Zhang H (2015). Beneficial effects of perioperative statins for major pulmonary resection. J Thorac Cardiovasc Surg.

[3] Durazzo AE, Machado FS, Ikeoka DT (2004). Reduction in cardiovascular events after vascular surgery with atorvastatin: a randomized trial. J Vasc Surg.

[4] Chopra V, Wesorick DH, Sussman JB (2012). Effect of perioperative statins on death, myocardial infarction, atrial fibrillation, and length of stay: a systematic review and meta-analysis. Arch Surg.

[5] de Waal BA, Buise MP, van Zundert AA (2015). Perioperative statin therapy in patients at high risk for cardiovascular morbidity undergoing surgery: a review. Br J Anaesth.

[6] Billings FT, Hendricks PA, Schildcrout JS (2016). High-dose perioperative atorvastatin and acute kidney injury following cardiac surgery: a randomized clinical trial. JAMA.

[7] Zheng Z, Jayaram R, Jiang L (2016). Perioperative rosuvastatin in cardiac surgery. N Engl J Med.

[8] Liberati A, Altman DG, Tetzlaff J (2009). The PRISMA statement for reporting systematic reviews and meta-analyses of studies that evaluate healthcare interventions: explanation and elaboration. BMJ.

[9] Higgins JPT, Green S (editors). Cochrane handbook for systematic reviews of interventions version 5.1.0 (updated March 2011). The Cochrane Collaboration, 2011. www.handbook.cochrane.org. Accessed 25 Sept 2018.

[10] Putzu A, Landoni G, Hajjar LA. Perioperative statin therapy: a systematic review, meta-analysis and trial sequential analysis of randomized controlled trials. PROSPERO 2018 CRD42018093997. http://www.crd.york.ac.uk/PROSPERO/display_record.php?ID=CRD42018093997. Accessed 25 Sept 2018.

[11] Brok J, Thorlund K, Gluud C, Wetterslev J (2008). Trial sequential analysis reveals insufficient information size and potentially false positive results in many meta-analyses. J Clin Epidemiol.

[12] Thorlund K, Devereaux PJ, Wetterslev J (2009). Can trial sequential monitoring boundaries reduce spurious inferences from meta-analyses?. Int J Epidemiol.

[13] Thorlund K, Engstrøm J, Wetterslev J, Brok J, Imberger G, Gluud C. User manual for trial sequential analysis (TSA). Copenhagen Trial Unit, Centre for Clinical Intervention Research, Copenhagen, Denmark. 2011. p. 1–115. www.ctu.dk/tsa. Accessed 25 Sept 2018.

[14] Almansob MA, Xu B, Zhou L (2012). Simvastatin reduces myocardial injury undergoing noncoronary artery cardiac surgery: a randomized controlled trial. Arterioscler Thromb Vasc Biol.

[15] Aydin U, Yilmaz M, Duzyol C (2015). Efficiency of postoperative statin treatment for preventing new-onset postoperative atrial fibrillation in patients undergoing isolated coronary artery bypass grafting: a prospective randomized study. Anatol J Cardiol.

[16] Baran C, Durdu S, Dalva K (2012). Effects of preoperative short term use of atorvastatin on endothelial progenitor cells after coronary surgery: a randomized, controlled trial. Stem Cell Rev.

[17] Bass AR, Szymonifka JD, Rondina MT (2018). Postoperative myocardial injury and inflammation is not blunted by a trial of atorvastatin in orthopedic surgery patients. HSS J.

[18] Berkan O, Katrancioglu N, Ozker E, Ozerdem G, Bakici Z, Yilmaz MB (2009). Reduced P-selectin in hearts pretreated with fluvastatin: a novel benefit for patients undergoing open heart surgery. Thorac Cardiovasc Surg.

[19] Berwanger O, de Barros ESPG, Barbosa RR (2017). Atorvastatin for high-risk statin-naive patients undergoing noncardiac surgery: the lowering the risk of operative complications using atorvastatin loading dose (LOAD) randomized trial. Am Heart J.

[20] Carrascal Y, Arnold RJ, De la Fuente L (2016). Efficacy of atorvastatin in prevention of atrial fibrillation after heart valve surgery in the PROFACE trial (PROphylaxis of postoperative atrial Fibrillation After Cardiac surgEry). J Arrhythm.

[21] Castano M, Gonzalez-Santos JM, Lopez J (2015). Effect of preoperative oral pravastatin reload in systemic inflammatory response and myocardial damage after coronary artery bypass grafting. A pilot double-blind placebo-controlled study. J Cardiovasc Surg (Torino).

[22] Chello M, Patti G, Candura D (2006). Effects of atorvastatin on systemic inflammatory response after coronary bypass surgery. Crit Care Med.

[23] Christenson JT (1999). Preoperative lipid-control with simvastatin reduces the risk of postoperative thrombocytosis and thrombotic complications following CABG. Eur J Cardiothorac Surg.

[24] Dehghani MR, Kasianzadeh M, Rezaei Y, Sepehrvand N (2015). Atorvastatin reduces the incidence of postoperative atrial fibrillation in statin-naive patients undergoing isolated heart valve surgery: a double-blind, placebo-controlled randomized trial. J Cardiovasc Pharmacol Ther.

[25] Hua P, Liu J, Tao J (2017). Efficacy and mechanism of preoperative simvastatin therapy on myocardial protection after extracorporeal circulation. Biomed Res Int.

[26] Ji Q, Mei Y, Wang X (2009). Effect of preoperative atorvastatin therapy on atrial fibrillation following off-pump coronary artery bypass grafting. Circ J.

[27] Mannacio VA, Iorio D, De Amicis V, Di Lello F, Musumeci F (2008). Effect of rosuvastatin pretreatment on myocardial damage after coronary surgery: a randomized trial. J Thorac Cardiovasc Surg.

[28] Mansour H, Ghaleb R (2017). Atorvastatin for reduction of postoperative atrial fibrillation in patients undergoing cardiac surgery. Atheroscler Suppl.

[29] Neilipovitz DT, Bryson GL, Taljaard M (2012). STAR VaS–short term atorvastatin regime for vasculopathic subjects: a randomized placebo-controlled trial evaluating perioperative atorvastatin therapy in noncardiac surgery. Can J Anaesth.

[30] Park JH, Shim JK, Song JW, Soh S, Kwak YL (2016). Effect of atorvastatin on the incidence of acute kidney injury following valvular heart surgery: a randomized, placebo-controlled trial. Intensive Care Med.

[31] Patti G, Chello M, Candura D (2006). Randomized trial of atorvastatin for reduction of postoperative atrial fibrillation in patients undergoing cardiac surgery: results of the ARMYDA-3 (Atorvastatin for Reduction of MYocardial Dysrhythmia After cardiac surgery) study. Circulation.

[32] Prowle JR, Calzavacca P, Licari E (2012). Pilot double-blind, randomized controlled trial of short-term atorvastatin for prevention of acute kidney injury after cardiac surgery. Nephrology (Carlton).

[33] Shyamsundar M, McAuley DF, Shields MO (2014). Effect of simvastatin on physiological and biological outcomes in patients undergoing esophagectomy: a randomized placebo-controlled trial. Ann Surg.

[34] Singh PP, Lemanu DP, Soop M, Bissett IP, Harrison J, Hill AG (2016). Perioperative simvastatin therapy in major colorectal surgery: a prospective, double-blind randomized controlled trial. J Am Coll Surg.

[35] Spadaccio C, Pollari F, Casacalenda A (2010). Atorvastatin increases the number of endothelial progenitor cells after cardiac surgery: a randomized control study. J Cardiovasc Pharmacol.

[36] Sun Y, Ji Q, Mei Y (2011). Role of preoperative atorvastatin administration in protection against postoperative atrial fibrillation following conventional coronary artery bypass grafting. Int Heart J.

[37] Tamayo E, Alvarez FJ, Alonso O (2009). Effects of simvastatin on systemic inflammatory responses after cardiopulmonary bypass. J Cardiovasc Surg (Torino).

[38] Vukovic PM, Maravic-Stojkovic VR, Peric MS (2011). Steroids and statins: an old and a new anti-inflammatory strategy compared. Perfusion.

[39] Xia J, Qu Y, Shen H, Liu X (2014). Patients with stable coronary artery disease receiving chronic statin treatment who are undergoing noncardiac emergency surgery benefit from acute atorvastatin reload. Cardiology.

[40] Youn YN, Park SY, Hwang Y, Joo HC, Yoo KJ (2011). Impact of high-dose statin pretreatment in patients with stable angina during off-pump coronary artery bypass. Korean J Thorac Cardiovasc Surg.

[41] Parepa IR, Suceveanu AI, Mazilu L, Mohamed A, Nita D, Tuta LA (2017). Preventing cardiac complications after non-cardiac non-vascular surgery by using perioperative statin therapy. Farmacia..

[42] Song YB, On YK, Kim JH (2008). The effects of atorvastatin on the occurrence of postoperative atrial fibrillation after off-pump coronary artery bypass grafting surgery. Am Heart J.

[43] Xia J, Qu Y, Yin C, Xu D (2015). Preoperative rosuvastatin protects patients with coronary artery disease undergoing noncardiac surgery. Cardiology.

[44] Nakamura K, Masuda H, Kariyazono H (2006). Effects of atorvastatin and aspirin combined therapy on inflammatory responses in patients undergoing coronary artery bypass grafting. Cytokine.

[45] Sanders RD, Nicholson A, Lewis SR, Smith AF, Alderson P (2013). Perioperative statin therapy for improving outcomes during and after noncardiac vascular surgery. Cochrane Database Syst Rev.

[46] Putzu A, Capelli B, Belletti A (2016). Perioperative statin therapy in cardiac surgery: a meta-analysis of randomized controlled trials. Crit Care.

[47] Juo YY, Mantha A, Ebrahimi R, Ziaeian B, Benharash P (2017). Incidence of myocardial infarction after high-risk vascular operations in adults. JAMA Surg.

[48] Devereaux PJ, Xavier D, Pohue J (2011). Characteristics and short-term prognosis of perioperative myocardial infarction in patients undergoing noncardiac surgery: a cohort study. Ann Intern Med.

[49] Almuti K, Rimawi R, Spevack D, Ostfeld RJ (2006). Effects of statins beyond lipid lowering: potential for clinical benefits. Int J Cardiol.

[50] Bellomo R (2016). Perioperative statins in cardiac surgery and acute kidney injury. JAMA.

[51] Golomb BA, Evans MA (2008). Statin adverse effects: a review of the literature and evidence for a mitochondrial mechanism. Am J Cardiovasc Drugs.

[52] Kain V, Kapadia B, Misra P, Saxena U (2015). Simvastatin may induce insulin resistance through a novel fatty acid mediated cholesterol independent mechanism. Sci Rep.

[53] Frieden TR (2017). Evidence for health decision making—beyond randomized, controlled trials. N Engl J Med.

[54] Baiardo Redaelli M, Landoni G, Di Sanzo S (2017). Interventions affecting mortality in critically ill and perioperative patients: a systematic review of contemporary trials. J Crit Care.

