# Supplementary material for: Perioperative statin therapy in cardiac and non-cardiac surgery: a systematic review and meta-analysis of randomized controlled trials
Source: Ann Intensive Care. 2018 Sep 27;8:95. doi: 10.1186/s13613-018-0441-3 (PMC6160380; doi:10.1186/s13613-018-0441-3)
Supplement: Supplementary file 1 — Additional file 1: Table 1. PRISMA 2009 checklist. Table 2. Major exclusions. Table 3. Further characteristics of the included trial. Figure 1. Risk of bias graph. Figure 2. Funnel plot for myocardial infarction. Figure 3. Funnel plot for stroke. Figure 4. Funnel plot for acute kidney injury. Figure 5. Funnel plot for mortality. Figure 6. Trial sequential analysis for postoperative myocardial infarction. Table 4. Primary and secondary analyses. Table 5. Sensitivity analyses. Table 6. Postoperative outcomes in statin-naïve trials. [file 13613_2018_441_MOESM1_ESM.doc]

**ADDITIONAL FILE 1**

**Perioperative statin therapy in cardiac and non-cardiac surgery:**

**a systematic review and meta-analysis of randomized controlled trials**

**AUTHORS**

Alessandro Putzu, Carolina Maria Pinto Domingues de Carvalho e Silva, Juliano Pinheiro de Almeida, Alessandro Belletti, Tiziano Cassina, Giovanni Landoni , Ludhmila Abrahao Hajjar

**SUMMARY**

eTable 1 - PRISMA 2009 Checklist [2](#__RefHeading___Toc397197721)

eMethods 1 - Search strategy for PubMed [4](#__RefHeading___Toc397197722)

eMethods 2 - Bias Risk Assessment [5](#__RefHeading___Toc397197723)

eTable 2 - Major Exclusions [6](#__RefHeading___Toc397197724)

eTable 3 - Further Characteristics of the included trial [8](#__RefHeading___Toc397197725)

eFigure 1 - Risk of bias graph [12](#__RefHeading___Toc397197726)

eFigure 2 – Funnel plot for myocardial infarction [13](#__RefHeading___Toc397197727)

eFigure 3 – Funnel plot for stroke [13](#__RefHeading___Toc397197728)

eFigure 4 – Funnel plot for acute kidney injury [14](#__RefHeading___Toc397197729)

eFigure 5 – Funnel plot for mortality [14](#__RefHeading___Toc397197730)

eResults 1 - Influence of conflicts of interest/funding on clinical outcomes [15](#__RefHeading___Toc397197731)

eFigure 6 - Trial sequential analysis for postoperative myocardial infarction [16](#__RefHeading___Toc397197732)

eTable 4 – Primary and secondary analyses [18](#__RefHeading___Toc397197733)

eTable 5 – Sensitivity analyses. [21](#__RefHeading___Toc397197734)

eTable 6 - Postoperative outcomes in statin-naïve trials. [23](#__RefHeading___Toc397197735)

eResults 2 - Influence of statin regimen on clinical outcomes [25](#__RefHeading___Toc397197736)

# eTable 1 - PRISMA 2009 Checklist

| **Section/topic** | **#** | **Checklist item** | **Reported on page #** |
| --- | --- | --- | --- |
| **TITLE** | | |  |
| Title | 1 | Identify the report as a systematic review, meta-analysis, or both. | 1 |
| **ABSTRACT** | | |  |
| Structured summary | 2 | Provide a structured summary including, as applicable: background; objectives; data sources; study eligibility criteria, participants, and interventions; study appraisal and synthesis methods; results; limitations; conclusions and implications of key findings; systematic review registration number. | 1 |
| **INTRODUCTION** | | |  |
| Rationale | 3 | Describe the rationale for the review in the context of what is already known. | 2 |
| Objectives | 4 | Provide an explicit statement of questions being addressed with reference to participants, interventions, comparisons, outcomes, and study design (PICOS). | 2 |
| **METHODS** | | |  |
| Protocol and registration | 5 | Indicate if a review protocol exists, if and where it can be accessed (e.g., Web address), and, if available, provide registration information including registration number. | 2 |
| Eligibility criteria | 6 | Specify study characteristics (e.g., PICOS, length of follow-up) and report characteristics (e.g., years considered, language, publication status) used as criteria for eligibility, giving rationale. | 2 |
| Information sources | 7 | Describe all information sources (e.g., databases with dates of coverage, contact with study authors to identify additional studies) in the search and date last searched. | 2 |
| Search | 8 | Present full electronic search strategy for at least one database, including any limits used, such that it could be repeated. | 2, Supplemental |
| Study selection | 9 | State the process for selecting studies (i.e., screening, eligibility, included in systematic review, and, if applicable, included in the meta-analysis). | 2 |
| Data collection process | 10 | Describe method of data extraction from reports (e.g., piloted forms, independently, in duplicate) and any processes for obtaining and confirming data from investigators. | 2 |
| Data items | 11 | List and define all variables for which data were sought (e.g., PICOS, funding sources) and any assumptions and simplifications made. | 2 |
| Risk of bias in individual studies | 12 | Describe methods used for assessing risk of bias of individual studies (including specification of whether this was done at the study or outcome level), and how this information is to be used in any data synthesis. | 2 |
| Summary measures | 13 | State the principal summary measures (e.g., risk ratio, difference in means). | 3 |
| Synthesis of results | 14 | Describe the methods of handling data and combining results of studies, if done, including measures of consistency (e.g., I^2^) for each meta-analysis. | 3 |
| Risk of bias across studies | 15 | Specify any assessment of risk of bias that may affect the cumulative evidence (e.g., publication bias, selective reporting within studies). | 3 |
| Additional analyses | 16 | Describe methods of additional analyses (e.g., sensitivity or subgroup analyses, meta-regression), if done, indicating which were pre-specified. | 3 |
| **RESULTS** | | |  |
| Study selection | 17 | Give numbers of studies screened, assessed for eligibility, and included in the review, with reasons for exclusions at each stage, ideally with a flow diagram. | 3, Figure 1 |
| Study characteristics | 18 | For each study, present characteristics for which data were extracted (e.g., study size, PICOS, follow-up period) and provide the citations. | 3, Table, Supplemental |
| Risk of bias within studies | 19 | Present data on risk of bias of each study and, if available, any outcome level assessment (see item 12). | 3, 4, Supplemental |
| Results of individual studies | 20 | For all outcomes considered (benefits or harms), present, for each study: (a) simple summary data for each intervention group (b) effect estimates and confidence intervals, ideally with a forest plot. | 4, 8-12, Supplemental |
| Synthesis of results | 21 | Present results of each meta-analysis done, including confidence intervals and measures of consistency. | _4, 8-12 Suppl_. |
| Risk of bias across studies | 22 | Present results of any assessment of risk of bias across studies (see Item 15). | _4, Suppl_. |
| Additional analysis | 23 | Give results of additional analyses, if done (e.g., sensitivity or subgroup analyses, meta-regression [see Item 16]). | _4, 8-12, Suppl_. |
| **DISCUSSION** | | |  |
| Summary of evidence | 24 | Summarize the main findings including the strength of evidence for each main outcome; consider their relevance to key groups (e.g., healthcare providers, users, and policy makers). | 9-12 |
| Limitations | 25 | Discuss limitations at study and outcome level (e.g., risk of bias), and at review-level (e.g., incomplete retrieval of identified research, reporting bias). | 12, 13 |
| Conclusions | 26 | Provide a general interpretation of the results in the context of other evidence, and implications for future research. | 13 |
| **FUNDING** | | |  |
| Funding | 27 | Describe sources of funding for the systematic review and other support (e.g., supply of data); role of funders for the systematic review. | 13 |

*From:*  Moher D, Liberati A, Tetzlaff J, Altman DG, The PRISMA Group (2009). Preferred Reporting Items for Systematic Reviews and Meta-Analyses: The PRISMA Statement. PLoS Med 6(7): e1000097. doi:10.1371/journal.pmed1000097

For more information, visit: **www.prisma-statement.org**.

# eMethods 1 - Search strategy for PubMed

(statin*[tiab] OR ((“hydroxymethylglutaryl-CoA reductase”[tiab] OR "HMGCoA reductase"[tiab]) AND inhibitor*[tiab]) OR anticholesteremic[tiab] OR simvastatin[tiab] OR rosuvastatin*[tiab] OR pravastatin*[tiab] OR atorvastatin*[tiab] OR fluvastatin*[tiab] OR cerivastatin*[tiab] OR pitavastatin*[tiab] OR lovastatin*[tiab]) AND (surgery[tiab] OR perioperative*[tiab] OR intervention*[tiab] OR “cardiac surgery”[tiab] OR “cardiovascular surgery”[tiab] OR “heart surgery”[tiab] OR “coronary artery bypass graft”[tiab] OR “valve replacement”[tiab] OR “valve repair”[tiab] OR “coronary surgery”[tiab]) AND (randomized controlled trial[pt] OR controlled clinical trial[pt] OR randomized controlled trials[mh] OR random allocation[mh] OR double-blind method[mh] OR single-blind method[mh] OR clinical trial[pt] OR clinical trials[mh] OR (clinical trial[tw] OR ((singl*[tw] OR doubl*[tw] OR trebl*[tw] OR tripl*[tw]) AND (mask*[tw] OR blind[tw])) OR (latin square[tw]) OR placebos[mh] OR placebo*[tw] OR random*[tw] OR research design[mh:noexp] OR follow-up studies[mh] OR prospective studies[mh] OR cross-over studies[mh] OR control*[tw] OR prospectiv*[tw] OR volunteer*[tw])) NOT (animal[mh] NOT human[mh])

# eMethods 2 - Bias Risk Assessment

We used the Cochrane methodology [1, 2] to evaluate this risk of bias of each included trials. Selection bias (random sequence generation and allocation concealment), performance bias (blinding of participants and investigators), detection bias (blinding of outcomes assessors), attrition bias (incomplete outcome data), reporting bias (selective outcome reporting), and other bias were judged to be of either low, unclear or high risk. The other bias domain included the classic items reported by the "Cochrane Handbook for Systematic Reviews of Interventions" [1, 2] but also the presence of: intention-to-treat analysis, sample size calculation, and ethical approval of the trial.

1. Higgins JP, Green S. Cochrane Handbook for Systematic Reviews of Interventions. Version 5. The Cochrane Collaboration, 2011; 2011.

2. Higgins JPT, Altman DG, Gøtzsche PC, Jüni P, Moher D, Oxman AD, et al. The Cochrane Collaboration’s tool for assessing risk of bias in randomised trials. BMJ. 2011;343:d5928.

# eTable 2 - Major Exclusions

| **Trial** | **Journal** | **Full-text** | **Reason for exclusion** |
| --- | --- | --- | --- |
| Alexander 2016 | Journal of Surgical Research | Full-text | Overlapping population |
| Almeida 2010 | Circulation | Abstract | Inappropriate design |
| Antoniades 2010 | Circulation | Full-text | Lack of outcome of interest |
| Antoniades 2012 | Journal of the American College of Cardiology | Full-text | Lack of outcome of interest |
| Berwanger 2015 | Circulation | Abstract | Overlapping population |
| Buyukbayrak 2013 | International Journal of Cardiology | Abstract | Lack of outcome of interest |
| Caorsi 2008 | Eur Cytokine Netw | Full-text | Lack of outcome of interest |
| Chee 2017 | Journal of Cardiothoracic Surgery | Full-text | Inappropriate design |
| Chello 2005 | European Journal of Cardio-thoracic Surgery | Full-text | Overlapping population |
| Chello 2007 | Ann Thorac Surg | Full-text | Lack of outcome of interest |
| Christenson 2001 | The American Journal of Cardiology | Full-text | Overlapping population |
| Cipollone 2003 | Circulation | Full-text | Retracted |
| Coccia 2007 | Clinical Therapeutics | Full-text | Lack of outcome of interest |
| Cortellaro 2002 | Thrombosis and Haemostasis | Abstract | Lack of outcome of interest |
| Crisby 2001 | Circulation | Full-text | Lack of outcome of interest |
| Cuccurullo 2006 | Arterioscler Thromb Vasc Biol | Full-text | Lack of outcome of interest |
| Dotani 2003 | The American Journal of Cardiology | Full-text | Lack of outcome of interest |
| Dunkelgrun 2009 (DECREASE IV) | Annals of Surgery | Full-text | Concerns about scientific integrity* |
| Evans 2007 | Eur J Vasc Endovasc Surg | Full-text | Lack of outcome of interest |
| Florens 2001 | J Card Surtg | Full-text | Lack of outcome of interest |
| Hong 2007 | The Journal of Urology | Full-text | Lack of outcome of interest |
| Hua 2017 | BioMed Research International | Full-text | Lack of outcome of interest |
| Kajimoto 2009 | Atherosclerosis | Full-text | Lack of outcome of interest |
| Kourliouros 2011 | The Journal of Thoracic and Cardiovascular Surgery | Full-text | Inappropriate design |
| Kumar 2013 | Indian Heart Journal | Abstract | Lack of outcome of interest |
| Ludman 2011 | Basic Res Cardiol | Full-text | Inappropriate design |
| Luijendijk 2014 | International Journal of Cardiology | Full-text | Inappropriate design |
| Makuuchi 2005 | Circ J | Full-text | Inappropriate design |
| Mansour 2016 | JACC: Cardiovascular Interventions | Abstract | Overlapping population |
| Martin-Ventura 2005 | Stroke | Full-text | Lack of outcome of interest |
| Masaki 2018 | General Thoracic and Cardiovascular Surgery | Full-text | Inappropriate design |
| Melina 2009 | Eur Hear J | Abstract | Lack of outcome of interest |
| Morantes Acevedo 2017 | Rev Med Hosp Gen Méx | Full-text | Lack of outcome of interest |
| Murtola 2017 | European Urology | Abstract | Lack of outcome of interest |
| Ozguler 2015 | Clin Invest Med | Full-text | Lack of outcome of interest |
| Parepa 2012 | European Journal of Preventive Cardiology | Abstract | Overlapping population |
| Pierri 2016 | General Thoracic and Cardiovascular Surgery | Full-text | Inappropriate design |
| Prowle 2010 | Intensive Care Medicine | Abstract | Overlapping population |
| Puato 2010 | Atherosclerosis Supplements | Abstract | Inappropriate design |
| Puato 2010 | Stroke | Full-text | Inappropriate design |
| Rahman 1995 | Annals of Vascular Surgery | Full-text | Lack of outcome of interest |
| Ramo 1995 | International Journal of Angiology | Full-text | Lack of outcome of interest |
| Saeed 2017 | Maedica – a Journal of Clinical Medicine | Full-text | Inappropriate design |
| Sarin 2016 | Updates Surg | Full-text | Lack of outcome of interest |
| Sasmazel 2008 | The Heart Surgery Forum | Full-text | Lack of outcome of interest |
| Schouten 2009 (DECREASE III) | The New England Journal of Medicine | Full-text | Concerns about scientific integrity* |
| Shokouhi 2014 | Jundishapur J Microbiol | Full-text | Lack of outcome of interest |
| Shyamsundar 2014 | Am J Respir Crit Care Med | Full-text | Lack of outcome of interest |
| Shyamsundar 2014 | Annals of Surgery | Full-text | Lack of outcome of interest |
| Singh 2016 | J Am Coll Surg | Full-text | Lack of outcome of interest |
| Tamayo 2007 | Med Clin (Barc) | Full-text | Overlapping population |
| Theilmeier 2011 | European Heart Journal | Abstract | Lack of outcome of interest |
| Zhou 2013 | Cardiology | Full-text | Lack of outcome of interest |

* according to previous reports (1-6).

1. Erasmus Medical College. Follow-up Investigation Committee. Investigation Into Possible Violation of Scientific Integrity: Report Summary. November 16, 2011.

2. Erasmus Medical College. Follow-up Investigation Committee. Report on the 2012 Follow-Up Investigation of Possible Breaches of Academic Integrity. September 30, 2012.

4. Erasmus Medical College. Follow-up Investigative Committee Academic Integrity 2013 July 25, 2014

3. Erasmus Medical College. Erasmus MC dismisses professor. Press release, November 2011.

5. Cole GD, Francis DP. Perioperative β blockade: guidelines do not reflect the problems with the evidence from the DECREASE trials. BMJ. 2014 Aug 29;349:g5210.

6. Sanders RD, Nicholson A, Lewis SR, Smith AF, Alderson P. Perioperative statin therapy for improving outcomes during and after noncardiac vascular surgery. Cochrane Database Syst Rev. 2013(7):CD009971.

# eTable 3 - Further Characteristics of the included trial

| **Trial** | **Design** | **Country** | **Endpoint of the study** | **Patients with chronic kidney disease excluded?** | **AKI definition** | **MI definition** | **Stroke definition** | **Intention-to-treat analysis** | **Risk of possible conflict of interests** | **Follow-up for mortality** |
| --- | --- | --- | --- | --- | --- | --- | --- | --- | --- | --- |
| **Cardiac surgery** | | | | | | | | |  |  |
| Almansob 2012 | Single center | China | Myocardial damage | Yes | NA | NR | NR | NR | Low | NR |
| Aydin 2015 | Single center | Turkey | AF | Yes | NA | NR | NR | NR | Unclear | 30 days |
| Baran 2011 | Single center | Turkey | Endothelial progenitor cells | Yes | Renal failure requiring dialysis | New Q wave in at least two leads or ST changes in association with significant troponin release. | NR | Yes | Low | 30-days |
| Berkan 2008 | Single center | Turkey | P-selectin | Yes | NA | New Q-waves or a reduction in R-waves > 25% in at least two leads. | NR | NR | Unclear | NA |
| Billings 2016 | Single center | USA | AKI | Yes | AKIN criteria | CK-MB elevation,  ECG changes,  echo wall motion abnormalities or reduced heart function, accompanied by troponin elevation | New neurological deficit with radiological evidence. | Yes | High | In-hospital |
| Carrascal 2016 | Single center | Spain | AF | Yes | Renal failure requiring dialysis | NR | NR | NR | Low | 30 months |
| Castaño 2015 | Single center | Spain | Myocardial damage and SIRS | No | NR | New Q-wave on ECG and CK-MB increase or regional wall motion abnormalities on echo. | NR | NR | High | 30-days |
| Chello 2006 | Single center | Italy | Cytokines | Yes | NR | NR | NR | Low | Unclear | NR |
| Christenson 1998 | Single center | Switzerland | Thrombocytosis and thrombotic complications | NR | Serum urea ≥ 9 mmol/l and serum creatinine ≥ 125 mmol/l in a patient with normal preoperative values. | New Q waves or significant loss of R-wave forces, with peak CK-MB > 10% of total CK. | NR | NR | High | In-hospital |
| Dehghani 2014 | Single center | Iran | AF | Yes | NA | NR | NR | Low | Low | In-hospital |
| Hua 2017 | Single center | China | Myocardial damage | Yes | NA | NA | NA | NR | Low | NR |
| Ji 2009 | Single center | China | AF | Yes | NA | NR | NR | Unclear | Unclear | In-hospital |
| Mannacio 2008 | Single center | Italy | Myocardial damage | Yes | Increase in serum creatinine value of greater than 2.5 mg/dL. | Increased biomarker with evidence of other change or echo evidence of new wall segments abnormalities. | NA | Unclear | Unclear | NR |
| Mansour 2016 | Single center | Egypt | AF | NR | NA | New Q waves or disappearance of R waves in at least 2 contiguous leads; elevated serum CK-MB activity 12 to 48 hours after surgery; and new wall segments abnormalities at echo. | New focal neurologic deficit lasting >24 hours with confirmation by CT or MRI scan. | Unclear | Unclear | NR |
| Nakamura 2006 | Single center | Japan | Inflammatory responses, endothelial function, and coagulation | Yes | NA | NA | NA | Unclear | Unclear | 14-days |
| Park 2016 | Single center | Korea | AKI | Yes | AKIN criteria | NA | NR | Yes | Low | In-hospital |
| Patti 2006 | Single center | Italy | AF | Yes | NA | New Q waves or a reduction in R waves  > 25% in at least 2 contiguous leads and elevated troponin levels at 12 hours. | NR | Unclear | Low | 30-days |
| Prowle 2012 | Single center | Australia | AKI | Yes | RIFLE criteria | NA | NA | Yes | High | In-hospital |
| Song 2008 | Single center | Korea | AF | Yes | NA | Two of the following criteria: new Q waves or disappearance of R waves in at least 2 contiguous leads; elevated CK-MB activity, and new wall segments abnormalities at echo. | New focal neurologic deficit > 24 hours with confirmation by CT or MRI scan. | Unclear | Unclear | NA |
| Spadaccio 2010 | Single center | Italy | Endothelial progenitor cells and cytokines | Yes | NR | NR | NR | NR | Low | NR |
| Sun 2011 | Single center | China | AF | Unclear | NA | NR | NR | NR | Unclear | NA |
| Tamayo 2009 | Single center | Spain | Cytokines | Yes | NA | NR | NR | Unclear | Unclear | NR |
| Vukovic 2010 | Single center | Serbia | Cardiac index | Yes | NA | NR | NA | Unclear | Unclear | NR |
| Youn 2011 | Single center | Korea | MACE: death from all causes, non-fatal myocardial infarction and repeat revascularization | Yes | NA | NR | NR | Unclear | Unclear | 30-days |
| Zheng 2016 | Single center | China | AF and myocardial injury | Yes | AKIN criteria | Third universal definition of myocardial infarction. | NR | Yes | High | In-hospital |
| **Non-cardiac surgery** | | | | | | | | | | |
| **Trial** | **Design** | **Country** | **Endpoint of the study** | **Patients with chronic kidney disease excluded?** | **AKI definition** | **MI definition** | **Stroke definition** | **Intention-to-treat analysis** | **Risk of possible conflict of interests** | **Follow-up for mortality** |
| Amar 2015 | Single-center | USA | Postoperative complications | NR | NA | NR | NA | Unclear | High | In-hospital |
| Bass 2018 | Single-center | USA | Myocardial injury | Yes | NA | NR | NR | Modified intention-to-treat analysis# | Low | 90-days |
| Berwanger 2017 | Multicenter | Brazil | Composite of all-cause mortality, nonfatal MINS, and stroke (MINS: elevated troponin judged to be due to cardiac ischemia). | No | NA | One of the following  1) histological confirmation necropsy; 2) new significant Q waves in at least 2 adjacent ECG leads; 3) New rise in troponin or CK-MB associated with one of the following: a) chest pain; b) typical ECG alterations; c) echo evidence or MIBI scan; d) need for urgent myocardial revascularization (PCI or CABG). | Focal neurologic deficit with imaging findings. | Yes | Low | 30 days |
| Durazzo 2004 | Single-center | Brazil | Composite of death from cardiac causes, nonfatal acute MI, ischemic stroke, and unstable angina. | Yes | NA | Elevated CK-MB or troponin associated with at least one of two: 12-lead ECG changes or evidence at autopsy | Signs or symptoms of ischemic stroke confirmed with imaging studies. | Yes | Low | 6 months |
| Neilipovitz 2012 | Single-center | Canada | C-reactive protein at 48 hours postoperatively | No | NA | Pathologic tissue findings or troponin increase and one of following: 1) ECG changes; 2) coronary artery intervention; 3) new echo regional wall motion abnormality; or 4) clinical correlation | NR | Yes | Low | 7 days |
| Parepa 2017 | Single-center | Romania | A composite endpoint of new-onset angina, silent cardiac ischemia, non-fatal acute coronary syndrome or fatal coronary heart disease. | NR | NA | NR | NA | Unclear | Unclear | 3-months |
| Shyamsundar 2014 | Single-center | UK | Dead space (Vd/Vt) at 6 hours after surgery | Yes | NA | NR | NA | Unclear | Unclear | NR |
| Singh 2016 | 4-centers | New Zealand | Postoperative complications by Clavien-Dindo classification system and Comprehensive  Complication Index. | Yes | NA | NR | NA | Yes | Unclear | 30-days |
| Xia 2014 | Single-center | China | Major adverse cardiac events: cardiac death, MI or unplanned revascularization | Yes | NR | New Q waves or a reduction in R waves >25% in at least 2 contiguous leads, regional wall motion abnormality, and clinical signs or symptoms together with increase in CK-MB or troponin levels to >3 times | NR | Yes | Low | 30-days |
| Xia 2015 | Single-center | China | Major adverse cardiovascular and cerebrovascular events: cardiac death, myocardial infarction, target vessel revascularization, stroke | Yes | NA | Ischemia due to a primary coronary event. | Permanent neurologic deficit sustained after a brain hemorrhage that was diagnosed by a neurologist and confirmed by imaging. | Yes | Low | 6 months |

AF, atrial fibrillation; MI, myocardial infarction; CK, creatine kinase; CK-MB, creatine kinase muscle/brain; AKIN criteria, Acute Kidney Injury Network criteria [1]; RIFLE, Risk, Injury, Failure, Loss of kidney function, and End-stage kidney disease [2]; GFR, glomerular filtration rate; NR, not reported; NA, not applicable; *Abstract-only publication. #, modified intention-to-treat analysis in which patients who received at least 1 dose of study drug (or placebo) postoperatively were included.

1. Mehta RL, Kellum JA, Shah S V, Molitoris BA, Ronco C, Warnock DG, et al. Acute Kidney Injury Network: report of an initiative to improve outcomes in acute kidney injury. Crit. Care. 2007;11:R31.

2. Bellomo R, Ronco C, Kellum JA, Mehta RL, Palevsky P, Acute Dialysis Quality Initiative workgroup. Acute renal failure - definition, outcome measures, animal models, fluid therapy and information technology needs: the Second International Consensus Conference of the Acute Dialysis Quality Initiative (ADQI) Group. Crit. Care. 2004;8:R204-12.

# eFigure 1 - Risk of bias graph: review authors' judgments about each risk of bias item presented as percentages across all included studies.


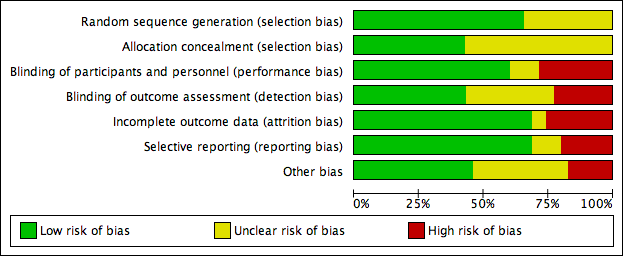


# eFigure 2 – Funnel plot for myocardial infarction


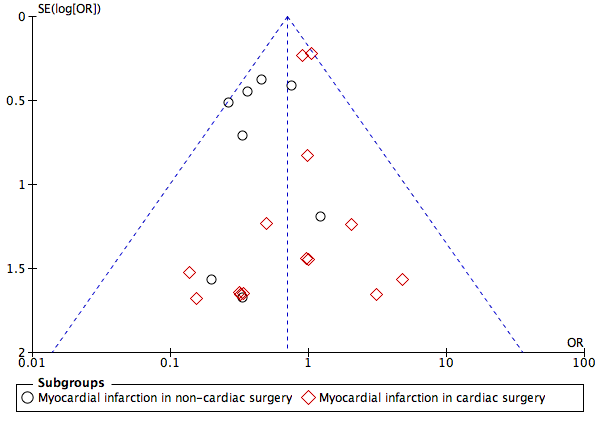


# eFigure 3 – Funnel plot for stroke

**
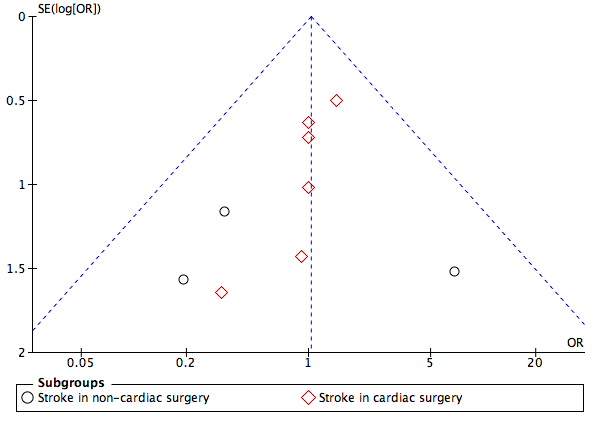
**

# eFigure 4 – Funnel plot for acute kidney injury


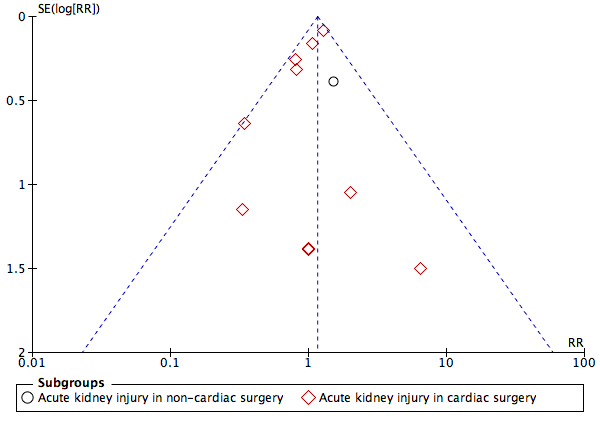


# eFigure 5 – Funnel plot for mortality


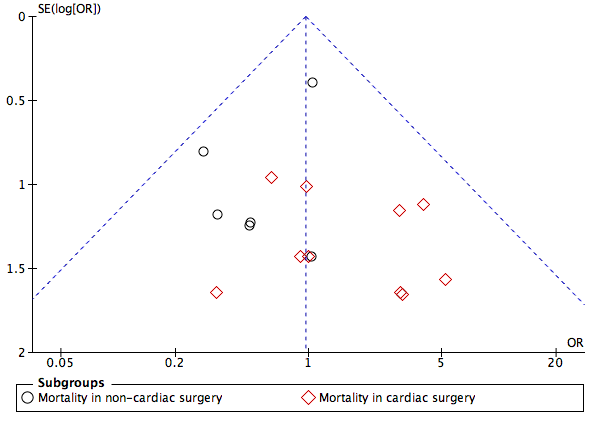


# eResults 1 - Influence of conflicts of interest/funding on clinical outcomes

Two authors (AP and AB or LAH) independently evaluated the presence of possible authors’ conflicts of interests and the funding of each study. In case of possible or unclear industrial conflicts of interest among studies included in the analysis, we performed sensitivity analysis excluding them and tested subgroup difference with the primary analysis.

Fourteen trials were judged to be at low risk of conflicts of interests, 14 at unclear risk, and 6 at higher risk (*see* eTable 3). No evidence of possible influence in favor of statins was found. Cardiac surgery: myocardial infarction (p_groups_=0.64), acute kidney injury (p_groups_=0.39), stroke (p_groups_=0.88), and mortality (p_groups_=0.87). Non-cardiac surgery: myocardial infarction (p_groups_=0.48), acute kidney injury (p_groups_=0.99), stroke (p_groups_=0.99), and mortality (p_groups_=0.49).

# eFigure 6 - Trial sequential analysis for postoperative myocardial infarction in non-cardiac surgery (A) and cardiac surgery (B).


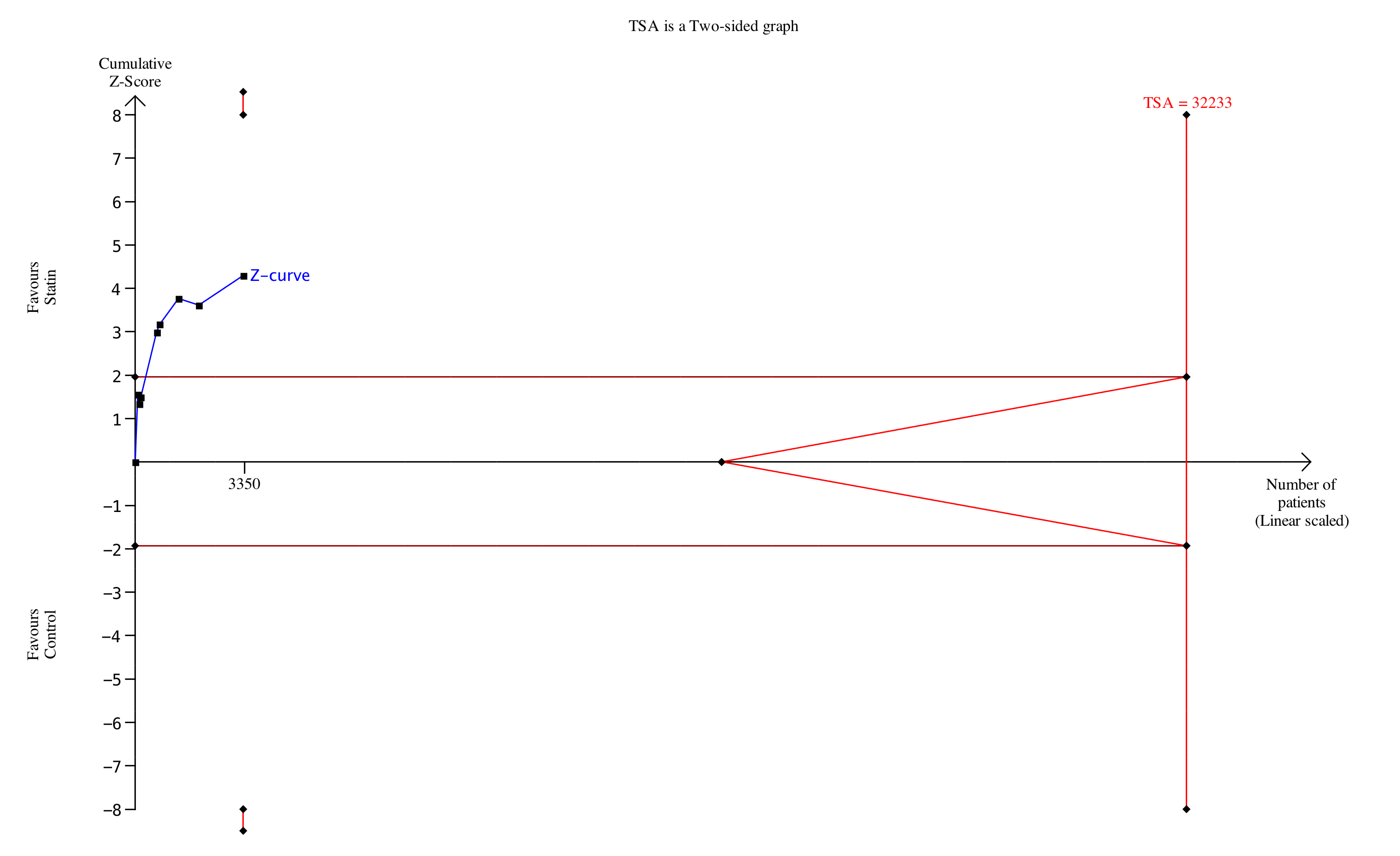
Trial sequential analysis of the included trials (black square fill icons) shows that the cumulative Z-curve did not cross nor the traditional boundary nor the trial sequential monitoring boundary for benefit nor for futility and did not reach the required information size (n=32233 and 3479 respectively), suggesting the need for more trials to establish firm conclusions about perioperative statins effects in non-cardiac surgery (A) and cardiac surgery (B). X-axis: the number of patients randomized; Y-axis: the cumulative Z-Score; Horizontal dotted lines: conventional boundaries (upper for benefit, Z-score = 1.96, lower for harm, Z-score = −1.96, two-sided, *p* value = 0.05); Oblique lines with diamond icons: trial sequential monitoring boundaries; Oblique line with square fill icons: Z-curve; Vertical line with diamonds: required information size. Risk of type I error = 5%, risk of type II error = 10% (power of 90%, relative risk reduction = 15%.

**A**


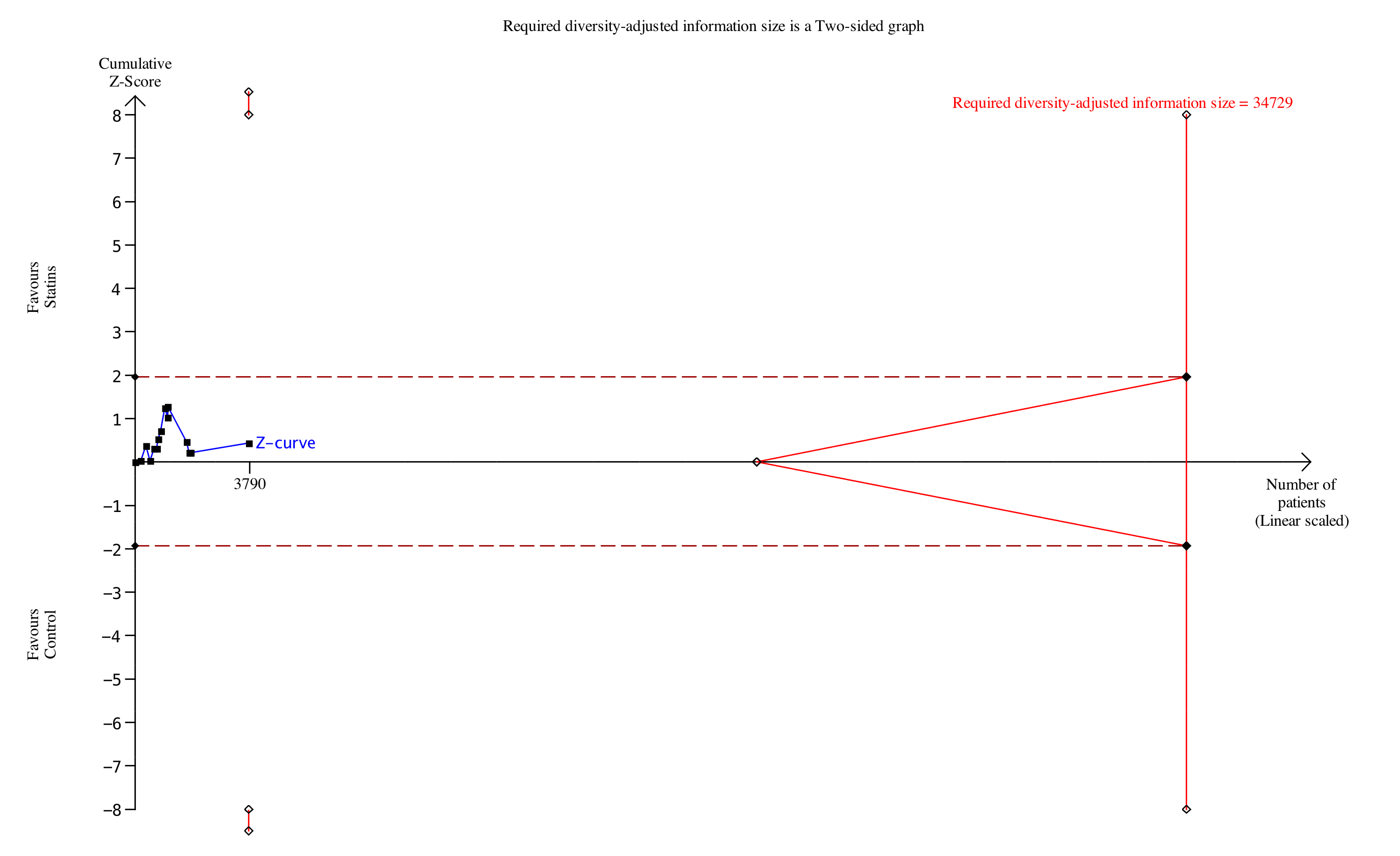
**B**

# eTable 4 – Primary and secondary analyses: effects of perioperative statin therapy versus control on postoperative outcomes.

| **Postoperative outcome** | **Number of trials** | **Number of patients** | **Effect estimate** | **P value** | **P value between groups** | **Trial sequential analysis**  **(TSA-adjusted confidence intervals)** |
| --- | --- | --- | --- | --- | --- | --- |
| Myocardial infarction  Cardiac surgery  - Low risk of bias trials  - High/unclear risk of bias trials  - All trials  Non cardiac surgery  - Low risk of bias trials  - High/unclear risk of bias trials  - All trials | 4  17  21  2  7  9 | 1388  1626  2208  300  2770  3370 | OR = 0.95 (95% CI, 0.70 to 1.30)  OR = 0.81 (95% CI, 0.39 to 1.65)  OR = 0.93 (95% CI, 0.70 to 1.24)  OR = 0.28 (95% CI, 0.13 to 0.64)  OR = 0.50 (95% CI, 0.32 to 0.77)  OR = 0.44 (95% CI, 0.30 to 0.64) | 0.77  0.56  0.61  *0.002*  *0.002*  < *0.001* | 0.68  0.23 | OR = 0.95 [TSA-adjusted 95% CI, 0.27 to 3.41]  -  OR = 0.93 [TSA-adjusted 95% CI, 0.29 to 3.01]  NA  -  OR = 0.44 [TSA-adjusted 95% CI, 0.09 to 2.07] |
| Stroke  Cardiac surgery  - Low risk of bias trials  - High/unclear risk of bias trials  - All trials  Non cardiac surgery  - Low risk of bias trials  - High/unclear risk of bias trials  - All trials | 4  7  13  1  5  6 | 1388  894  1840  50  1302  718 | OR = 1.19 (95% CI, 0.61 to 2.34)  OR = 0.75 (95% CI, 0.18 to 3.09)  OR = 1.09 (95% CI, 0.60 to 2.00)  OR = 0.19 (95% CI, 0.01 to 4.10)  OR = 1.27 (95% CI, 0.31 to 5.16)  OR = 0.82 (95% CI, 0.25 to 2.71) | 0.61  0.69  0.77  0.29  0.74  0.75 | 0.56  0.27 | OR = 1.19 [TSA-adjusted 95% CI, 0.43 to 3.28]  -  OR = 1.09 [TSA-adjusted 95% CI, 0.47 to 2.56]  NA  -  NA |
| Acute kidney injury  Cardiac surgery  - Low risk of bias trials  - High/unclear risk of bias trials  - All trials  Non cardiac surgery  - Low risk of bias trials  - High/unclear risk of bias trials  - All trials | 5  6  11  0  3  3 | 1438  517  1700  -  115  115 | RR = 1.17 (95% CI, 1.02 to 1.34)  RR = 0.67 (95% CI, 0.30 to 1.49)  RR = 1.15 (95% CI, 1.00 to 1.31)  -  RR =1.52 (95% CI, 0.71 to 3.26)  RR =1.52 (95% CI, 0.71 to 3.26) | *0.03*  0.32  *0.05*  -  0.28  0.28 | 0.18  - | RR = 1.17 [TSA-adjusted 95% CI, 0.84 to 1.62]  -  RR = 1.15 [TSA-adjusted 95% CI, 0.87 to 1.50]  -  -  NA |
| Mortality  Cardiac surgery  - Low risk of bias trials  - High/unclear risk of bias trials  - All trials  Non cardiac surgery  - Low risk of bias trials  - High/unclear risk of bias trials  - All trials | 5  17  22  3  7  10 | 1438  1561  2242  365  2770  3502 | OR = 3.71 (95% CI, 1.03 to 13.34)  OR = 0.87 (95% CI, 0.33 to 2.30)  OR = 1.59 (95% CI, 0.76 to 3.31)  OR = 0.60 (95% CI, 0.14 to 2.54)  OR = 0.72 (95% CI, 0.38 to 1.36)  OR = 0.70 (95% CI, 0.39 to 1.25) | *0.04*  0.78  0.22  0.49  0.31  0.22 | 0.08  0.82 | OR = 3.71 [TSA-adjusted 95% CI, 0.02 to 690]  -  OR = 1.59 [TSA-adjusted 95% CI, 0.08 to 31.6]  NA  -  NA |
| Need of renal replacement therapy  Cardiac surgery  - Low risk of bias trials  - High/unclear risk of bias trials  - All trials  Non cardiac surgery  - Low risk of bias trials  - High/unclear risk of bias trials  - All trials | 5  2  7  0  0  0 | 2867  150  3017 | OR = 1.28 [95% CI, 0.64 to 2.54]  OR = 6.84 [95% CI, 0.34 to 136.41]  OR = 1.46 [95% CI, 0.75 to 2.81] | 0.26  0.21  0.26 | 0.28 | NA  -  NA |
| Acute kidney injury not requiring renal replacement therapy  Cardiac surgery  - Low risk of bias trials  - High/unclear risk of bias trials  - All trials  Non cardiac surgery  - Low risk of bias trials  - High/unclear risk of bias trials  - All trials | 5  0  5 | 2867  -  2867 | OR = 1.22 [95% CI, 1.02 to 1.46]  OR = 1.22 [95% CI, 1.02 to 1.46] | *0.03*  *0.03* |  | OR = 1.22 [TSA-adjusted 95% CI, 0.85 to 1.75]  OR = 1.22 [TSA-adjusted 95% CI, 0.85 to 1.75] |

OR, odds ratio; RR, risk ratio; CI, confidence interval; NA, not applicable (analysis not possible due too little information size).

# eTable 5 – Sensitivity analyses.

| **Postoperative outcome** | **Effect estimate** | **P value** |
| --- | --- | --- |
| Myocardial infarction  Cardiac surgery  - Primary analysis  - Relative Risk  - Risk difference  - Random effects model  Non cardiac surgery  - Primary analysis  - Relative Risk  - Risk difference  - Random effects model | OR = 0.93 (95% CI, 0.70 to 1.24)  RR = 0.94 (95% CI, 0.72 to 1.21)  RD = -0.00 (95% CI, -0.02 to 0.01)  OR = 0.94 (95% CI, 0.70 to 1.26)  OR = 0.44 (95% CI, 0.30 to 0.64)  RR = 0.45 (95% CI, 0.31 to 0.65)  RD = -0.03 (95% CI, -0.04 to -0.02)  OR = 0.44 (95% CI, 0.30 to 0.66) | 0.61  0.61  0.64  0.68  < *0.001*  < *0.001*  < *0.001*  < *0.001* |
| Stroke  Cardiac surgery  - Primary analysis  - Relative Risk  - Risk difference  - Random effects model  Non cardiac surgery  - Primary analysis  - Relative Risk  - Risk difference  - Random effects model | OR = 1.09 (95% CI, 0.60 to 2.00)  RR = 1.09 (95% CI, 0.60 to 1.97)  RD = 0.00 (95% CI, -0.01 to 0.01)  OR = 1.10 (95% CI, 0.60 to 2.03)  OR = 0.82 (95% CI, 0.25 to 2.71)  RR = 0.82 (95% CI, 0.25 to 2.70)  RD = -0.00 (95% CI, -0.01 to 0.01)  OR = 0.71 (95% CI, 0.09 to 5.64) | 0.77  0.77  0.79  0.76  0.75  0.75  0.78  0.74 |
| Acute kidney injury  Cardiac surgery  - Primary analysis  - Odds Ratio  - Risk difference  - Random effects model  Non cardiac surgery  - Primary analysis  - Odds Ratio  - Risk difference  - Random effects model | RR = 1.15 (95% CI, 1.00 to 1.31)  OR = 1.19 (95% CI, 1.00 to 1.41)  RD = 0.03 (95% CI, -0.00 to 0.05)  RR = 1.05 (95% CI, 0.85 to 1.30)  RR =1.52 (95% CI, 0.71 to 3.26)  OR =1.62 (95% CI, 0.68 to 3.86)  RD = 0.04 (95% CI, -0.04 to 0.12)  RR =1.52 (95% CI, 0.71 to 3.26) | *0.05*  *0.05*  *0.05*  0.65  0.28  0.28  0.30  0.28 |
| Mortality  Cardiac surgery  - Primary analysis  - Risk Ratio  - Risk difference  - Random effects model  Non cardiac surgery  - Primary analysis  - Risk Ratio  - Risk difference  - Random effects model | OR = 1.59 (95% CI, 0.76 to 3.31)  RR = 1.57 (95% CI, 0.77 to 3.20)  RD = 0.00 (95% CI, -0.00 to 0.01)  OR = 1.50 (95% CI, 0.68 to 3.31)  OR = 0.70 (95% CI, 0.39 to 1.25)  RR = 0.70 (95% CI, 0.40 to 1.24)  RD = -0.00 (95% CI, -0.01 to 0.00)  OR = 0.73 (95% CI, 0.40 to 1.33) | 0.22  0.22  0.29  0.32  0.22  0.23  0.24  0.30 |

CI, confidence interval; OR, odds ratio; RR, risk ratio; RD, risk difference.

# eTable 6 - Postoperative outcomes in statin-naïve trials.

| **Postoperative outcome** | **Statin-naïve** | **Chronic therapy/mixed population** |
| --- | --- | --- |
| Myocardial infarction  Cardiac surgery  - Low risk of bias trials  - All trials  Non cardiac surgery  - Low risk of bias trials  - All trials | 0 trials  OR = 0.98 (95% CI, 0.46 to 2.11), 15 trials (n = 1475)  OR = 0.34 (95% CI, 0.08 to 1.35), 1 trial (n = 100)  OR = 0.49 (95% CI, 0.30 to 0.81), 7 trials (n = 2320) | OR = 0.95 (95% CI, 0.70 to 1.30), 3 trials (n = 2567)  OR = 0.92 (95% CI, 0.68 to 1.25), 6 trials (n = 2918)  OR = 0.26 (95% CI, 0.10 to 0.72), 1 trial (n = 500)  OR = 0.37 (95% CI, 0.21 to 0.67), 2 trials (n = 1050) |
| Stroke  Cardiac surgery  - Low risk of bias trials  - All trials  Non cardiac surgery  - Low risk of bias trials  - All trials | 0 trials  OR = 0.88 (95% CI, 0.32 to 2.43), 9 trials (n = 962)  OR = 0.19 (95% CI, 0.01 to 4.10), 1 trial (n = 100)  OR = 1.17 (95% CI, 0.04 to 39.00), 5 trials (n = 852) | OR = 1.25 (95% CI, 0.58 to 2.70), 3 trials (n = 2567)  OR = 1.25 (95% CI, 0.58 to 2.70), 3 trials (n = 2567)  0 trials  OR = 0.33 (95% CI, 0.03 to 3.20), 1 trial (n = 550) |
| Acute kidney injury  Cardiac surgery  - Low risk of bias trials  - All trials  Non cardiac surgery  - Low risk of bias trials  - All trials | RR = 1.08 (95% CI, 0.73 to 1.59), 2 trials (n = 399)  RR = 1.09 (95% CI, 0.75 to 1.57), 7 trials (n = 839)  0 trials  RR =1.52 (95% CI, 0.71 to 3.26), 3 trials (n = 239) | RR = 1.18 (95% CI, 1.02 to 1.37), 4 trials (n = 2468)  RR = 1.16 (95% CI, 1.00 to 1.34), 5 trials (n = 2545)  0 trials  0 trials |
| Mortality  Cardiac surgery  - Low risk of bias trials  - All trials  Non cardiac surgery  - Low risk of bias trials  - All trials | 0 trials  OR = 1.11 (95% CI, 0.42 to 2.94), 14 trials (n = 1379)  OR = 0.67 (95% CI, 0.11 to 4.08), 2 trials (n = 232)  OR = 0.71 (95% CI, 0.39 to 1.30), 8 trials (n = 2452) | OR = 3.84 (95% CI, 0.95 to 15.55), 2 trials (n = 2667)  OR = 2.52 (95% CI, 0.79 to 8.06), 6 trials (n = 2840)  OR = 0.50 (95% CI, 0.04 to 5.53), 2 trials (n = 500)  OR = 0.50 (95% CI, 0.04 to 5.53), 2 trials (n = 1050) |

OR, odds ratio; RR, risk ratio; CI, confidence interval. No significant difference between groups were found (p_groups_ > 0.05).

#

# eResults 2 - Influence of statin regimen on clinical outcomes

Per-protocol fixed-effect meta-regression was employed to examine the possible influence of statin therapy regimen on clinical outcomes. We assessed the impact of length of preoperative statin therapy, length of postoperative statin therapy, total length of perioperative statin therapy (preoperative + postoperative), and proportion of statin-naïve patients. The meta-regression was performed using Open Meta-Analyst [1, 2].

The analyses failed to find any possible correlation between outcome and statin regimen variables. Cardiac surgery: myocardial infarction (*p* values: 0.65, 0.45, 0.86, 0.97 respectively), acute kidney injury (*p* values: 0.58, 0.07, 0.37, 0.95), stroke (*p* values: 0.73, 0.87, 0.74, 0.60), and mortality (*p* values: 0.48, 0.50, 0.80, 0.45). Non-cardiac surgery: myocardial infarction (*p* values: 0.68, 0.93, 0.99, 0.276), acute kidney injury (*p* values: 0.78, 0.82, 0.81, 0.29), stroke (*p* values: 0.92, 0.62, 0.66, 0.45), and mortality (*p* values: 0.19, 0.38, 0.27, 0.88).

1. Wallace BC, Dahabreh IJ, Trikalinos TA, Lau J, Trow P, Schmid CH. Closing the Gap between Methodologists and End-Users: R as a Computational Back-End. J. Stat. Softw. 2012;49:1–15.

2. Viechtbauer W. Conducting Meta-Analyses in R with the metafor Package. J. Stat. Softw. 2010;36:1–48.
